# Sialylated Immunoglobulin G Promotes the Malignant Progression of Oral Squamous Cell Carcinoma through VCP-Mediated NDUFB6 Stabilization Regulated Mitochondrial Oxidative Phosphorylation

**DOI:** 10.34133/research.0985

**Published:** 2025-12-12

**Authors:** Lin Qiu, Anqi Tao, Linlin Zhang, Yiheng Liao, Gang Zhao, Xiaoyan Qiu, Cuiying Li

**Affiliations:** ^1^ Central Laboratory, Peking University School and Hospital of Stomatology & National Center for Stomatology & National Clinical Research Center for Oral Diseases & National Engineering Research Center of Oral Biomaterials and Digital Medical Devices, Beijing, China.; ^2^ Department of Oral and Maxillofacial Surgery, Peking University School and Hospital of Stomatology & National Center for Stomatology & National Clinical Research Center for Oral Diseases & National Engineering Research Center of Oral Biomaterials and Digital Medical Devices, Beijing, China.; ^3^ Department of Periodontology, Peking University School and Hospital of Stomatology & National Center for Stomatology & National Clinical Research Center for Oral Diseases & National Engineering Research Center of Oral Biomaterials and Digital Medical Devices, Beijing, China.; ^4^ Department of Orthodontics, Stomatology College of Jiamusi University, Jiamusi, China.; ^5^ Department of Immunology, School of Basic Medical Sciences, Peking University, NHC Key Laboratory of Medical Immunology, Beijing, China.

## Abstract

The absence of distinct biomarkers for oral squamous cell carcinoma (OSCC) hampers clinical diagnosis and treatment. Previous studies have demonstrated that sialylated immunoglobulin G (SIA-IgG) is up-regulated in a variety of epithelial-derived tumors and plays an important biological role. However, the function of SIA-IgG in OSCC progression is yet to be clarified. The expression characteristics and clinical significance of SIA-IgG were investigated using immunohistochemistry, western blot, and bioinformatics analysis. The biological functions of SIA-IgG were evaluated in vitro and in vivo. The underlying mechanisms of SIA-IgG in OSCC were elucidated using immunoprecipitation, mass spectrometry, and proteomic profiling. Furthermore, we explored the inhibitory effects of an exogenously added SIA-IgG-specific monoclonal antibody, RP215, on OSCC. An elevated level of SIA-IgG was observed in OSCC, and this was linked to poor survival among OSCC patients. Meanwhile, the up-regulation of SIA-IgG and valosin-containing protein (VCP) promoted the malignant progression of OSCC by activating the oxidative phosphorylation pathway. Moreover, SIA-IgG showed potential as a marker of OSCC cancer stem cells. Mechanistically, SIA-IgG enhanced the interaction between VCP and NADH:ubiquinone oxidoreductase subunit B6 (NDUFB6), thereby inhibiting the ubiquitin–proteasome-pathway-mediated degradation of NDUFB6 and sustaining the activation of the oxidative phosphorylation pathway. This inhibitory effect was abrogated by the overexpression of VCP. Furthermore, the SIA-IgG-specific antibody RP215 suppressed the malignant progression of OSCC, both in vitro and in vivo.

## Introduction

Oral cancer has been recognized as a widespread cancer that occurs in the head and neck. In 2022, over 350,000 were newly diagnosed worldwide, with more than 175,000 deaths reported [1]. Over 90% of all such cases are oral squamous cell carcinoma (OSCC) [2]. Although the introduction of targeted therapy, immunotherapy [3], and other methods has made the comprehensive sequential treatment model for OSCC increasingly mature, the 5-year survival rate of patients with early-stage OSCC is roughly 60%, whereas it drops to approximately 30% for those with advanced-stage disease [4]. Consequently, the early diagnosis and inhibition of the malignant progression of OSCC remain focal areas of research in oral and maxillofacial oncology. By identifying the oncogenes implicated in OSCC and elucidating the critical molecular pathways and associated mechanisms underlying its initiation and progression, we can then find effective combinatorial therapeutic approaches, which represent an important avenue for resolving this clinical challenge.

Conventionally, B cells generate immunoglobulins, which function to defend the body from infections. However, emerging findings indicate that immunoglobulins exist in non-B cells as well, including neurons [5], spermatogenic cells [6], and hepatocytes [7]. Notably, elevated levels of immunoglobulin G (IgG) have been detected in epithelial-derived cancers such as pancreatic ductal adenocarcinoma [8], breast cancer [9], prostate cancer [10], and lung cancer [11]. This IgG is termed as cancer-derived IgG. Cancer-derived IgG retains the fundamental structure of IgG [12]. Our previous research has demonstrated that in addition to undergoing glycosylation at the classic asparagine (Asn) 297 site within the constant region of the C_H_2 domain [12], it is also glycosylated at the Asn162 site within the constant region of the C_H_1 domain [13]. Moreover, it is terminally modified with a high level of sialic acid, thus designated as sialylated IgG (SIA-IgG) [13]. The distinctive properties of SIA-IgG endow it with unique biological functions that play pivotal roles in stemness maintenance [11], cancer progression [13], and immune evasion [14]. Furthermore, owing to its specific modifications, the monoclonal antibody RP215 can specifically identify SIA-IgG [13]. Previous studies have indicated that, compared to that in normal oral squamous epithelium and salivary gland epithelium, elevated expression of SIA-IgG is observed in OSCC and pleomorphic adenoma [15]. Regrettably, research on SIA-IgG in the context of OSCC has been limited, and the significance and underlying mechanisms of its overexpression in OSCC remain unclear.

Metabolic reprogramming is a hallmark of cancer, enabling rapid growth and proliferation of cancer cells [16,17]. Accumulating evidence suggests that mitochondrial function remains largely intact in the majority of tumor cells. Moreover, various tumor cells, including OSCC, leukemia, lymphoma, and endometrial cancer, exhibit increased glycolysis and maintain functional oxidative phosphorylation (OXPHOS) [18]. OXPHOS not only provides sufficient energy for tumor cell survival but also regulates proliferation, invasion, and metastasis [19]. OXPHOS relies on a series of electron carriers and enzymes that form the electron transport chain (ETC) to increase electron affinity. The ETC comprises 4 complexes (I, II, III, and IV) that are involved in electron transfer. These complexes work in concert to generate a substantial amount of adenosine triphosphate (ATP), thereby supplying ample energy to the tumor cells [20]. NADH:ubiquinone oxidoreductase subunit B6 (NDUFB6), a crucial subunit of complex I, plays an indispensable role in the ETC process. It finely regulates the interactions and assembly among various subunits of complex I, ensuring the formation of a stable and fully functional structure that efficiently participates in ETC [21]. In recent years, studies have demonstrated that NDUFB6 is closely associated with the initiation and progression of multiple types of tumors, including stomach adenocarcinoma [22], acute myeloid leukemia [21], and colorectal cancer [23]. However, the specific mechanisms underlying the role of NDUFB6 in OXPHOS in OSCC cells remain unclear.

Valosin-containing protein (VCP), located on human chromosome 9p13.3, is a member of the ATPase family. It encompasses 2 ATPase domains, namely, the D1 and D2 regions, and its multidomain architecture endows it with the capacity to interact with a diverse array of proteins. Similar to other members of its family, VCP primarily functions as a molecular chaperone, facilitating protein folding and unfolding [24]. Research has demonstrated that VCP participates in multiple cellular processes, including autophagy, and protein folding [25,26]. Yamamoto et al. [27] analyzed 74 OSCC samples and found that the expression level of VCP in OSCC was significantly higher than that in normal tissues, and its expression was notably correlated with OSCC metastasis and recurrence. Furthermore, recent studies have indicated a close association between VCP expression and mitochondrial function [28]. The specific role of VCP and its relationship with OXPHOS in OSCC remain to be elucidated.

In this study, immunohistochemical examination of tumor tissue microarrays revealed SIA-IgG up-regulation within OSCC tissues. Notably, SIA-IgG expression was significantly related to OSCC patient survival, which was an independent prognostic indicator of individuals with this disease. As demonstrated by a series of experiments, SIA-IgG enhanced malignant progression of OSCC in vitro and in vivo and might be an OSCC cancer stem cell (CSC) marker. Furthermore, we discovered that SIA-IgG stabilizes NDUFB6 by binding to VCP, inhibiting the degradation of the ubiquitin–proteasome pathway of NDUFB6 and continuously activating the OXPHOS pathway to promote OSCC malignant progression. This study underscores the functional and clinical significance of SIA-IgG in OSCC, which may be the target for OSCC therapeutic interventions.

## Results

### In OSCC, the SIA-IgG level shows a positive relation to unfavorable outcomes and can function as an independent prognostic marker

**Fig. 1.**
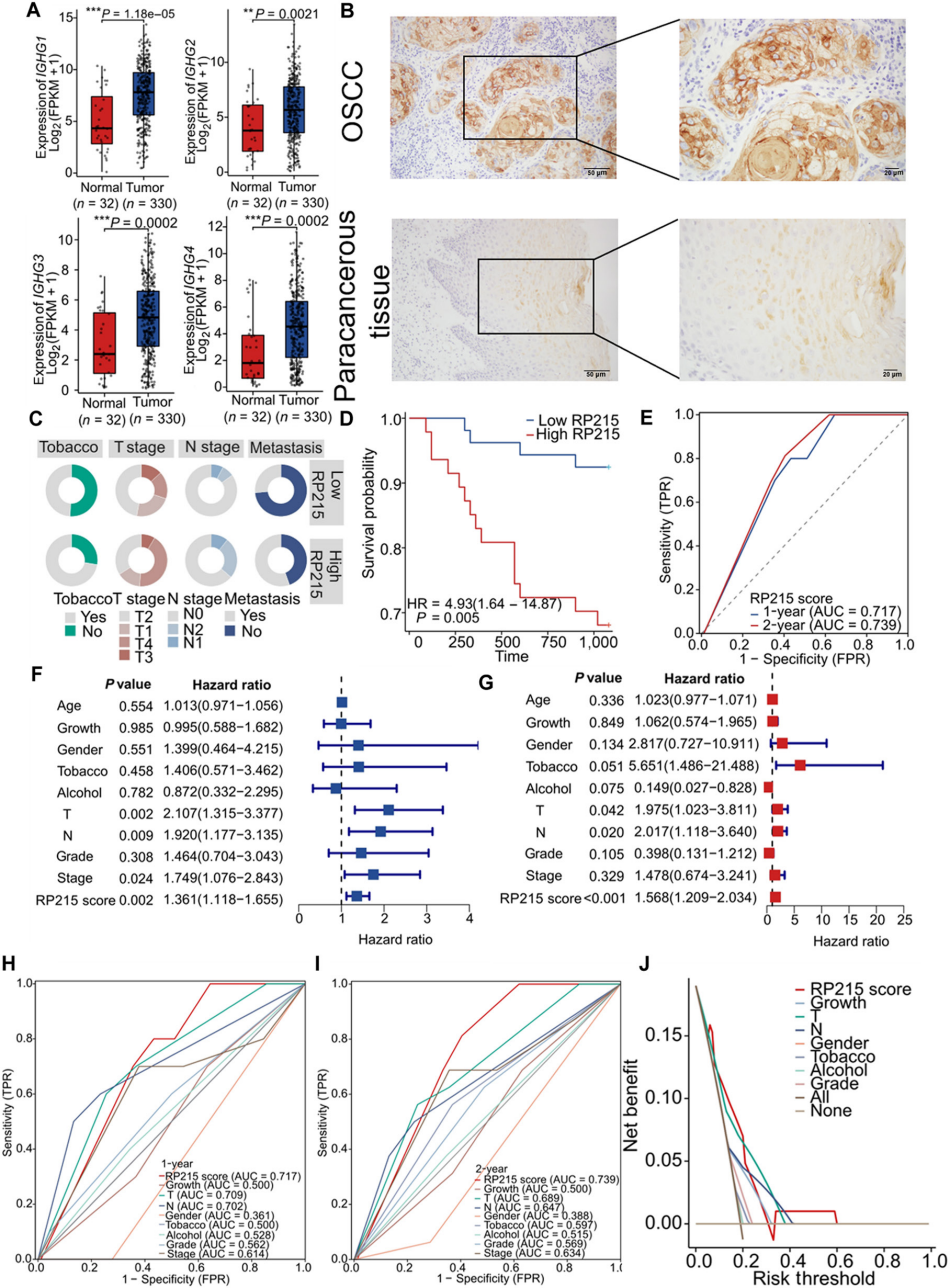
Increased sialylated immunoglobulin G (SIA-IgG) expression correlates positively with unfavorable prognosis and can be an independent prognostic factor of oral squamous cell carcinoma (OSCC). (A) Expression levels of *IGHG1*, *IGHG2*, *IGHG3*, and *IGHG4* in OSCC (*n* = 330) versus those in normal tissues (*n* = 32) from The Cancer Genome Atlas (TCGA) database (red indicates normal tissues; the blue column represents tumor tissues). (B) Typical immunohistochemistry (IHC) staining of SIA-IgG in paired OSCC and paracancerous tissues; *n* = 100 per group (left: magnification ×40, scale bar 50 μm; right: magnification ×100, scale bar 20 μm). (C) Pie distribution map of the clinicopathological characteristics of patients significantly related to SIA-IgG; *n* = 53 in the low-RP215 group, and *n* = 47 in the high-RP215 group. (D) Survival curves of OSCC cases showing high or low RP215 scores and analyzed by the log-rank test; *n* = 53 in the low-RP215 group, and *n* = 47 in the high-RP215 group. (E) The 1- and 2-year receiver operating characteristic (ROC) curves and areas under the curve (AUCs); *n* = 100 per group. (F) Univariate and multivariate Cox regression (G) analysis; *n* = 100 per group. (H to J) One-year multifactorial ROC curves, 2-year multifactorial ROC curves, and decision curve analysis (DCA) curves based on RP215 staining scores and clinicopathological characteristics, respectively; *n* = 100 per group. **P < 0.01 and ***P < 0.001 by unpaired *t* test. FPKM, fragments per kilobase of exon model per million mapped fragments; HR, hazard ratio; TPR, true positive rate; FPR, false positive rate.

For investigating IgG expression in OSCC, our first step was to analyze the IgG messenger RNA levels of OSCC (*n* = 330) and normal tissues (*n* = 32) in The Cancer Genome Atlas (TCGA) database, revealing a significant increase in IgG expression in OSCC compared with that in normal tissues (Fig. 1A). Consistent results were obtained by retrieving immunohistochemistry (IHC) images of IgG in the normal oral mucosa and OSCC tissues from the Human Protein Atlas database (Fig. S1A). Subsequently, we performed IHC staining and western blot to assess the expression of SIA-IgG in paired tissue samples. The IHC results indicated higher expression levels of SIA-IgG in cancer cells than in normal epithelial cells (Fig. 1B). Western blot results also demonstrated up-regulated expression of SIA-IgG in OSCC tissues compared to that in normal tissues (Fig. S1B). Moreover, SIA-IgG up-regulation could be observed within multiple OSCC cells (Fig. S1C and D), suggesting that SIA-IgG may act as an oncogene in OSCC. Integrating the RP215 IHC scores with the clinicopathological information of OSCC patients suggested that SIA-IgG level was significantly related to patients’ smoking habits and TNM stage (Fig. 1C and Table 1). Sorting patients based on IHC scores demonstrated that those with high scores had poorer survival status and a higher likelihood of death (Fig. S1E). From further survival analysis, those having increased SIA-IgG expression showed significantly decreased 3-year overall survival (OS) rates compared to those with low SIA-IgG expression (Fig. 1D). Meanwhile, based on receiver operating characteristic (ROC) curves, the areas under the curve for predicting the 1- and 2-year OS rates of OSCC patients using IHC scores were 0.717 and 0.739, respectively (Fig. 1E), indicating the high predictive efficacy of RP215 IHC scores for OSCC patient prognosis. According to univariate and multivariate Cox regression results, the RP215 IHC score was the independent factor for predicting OSCC prognosis (Fig. 1F and G). Subsequently, the multivariate ROC curve and decision curve analysis curve both indicated that the RP215 IHC score was a superior prognostic indicator compared to the other clinicopathological features (Fig. 1H to J). According to these results, the prognostic nomogram and its internal calibration curve were established, which demonstrated relatively accurate predictions of 1- and 2-year survival rates for OSCC patients (Fig. S1F and G). Collectively, SIA-IgG expression increases within OSCC, which is positively related to poor patient prognosis and is an independent factor for forecasting OSCC prognosis.

**Table 1.** Relations of SIA-IgG with clinicopathological features for OSCC patients. The bold clinicopathological features indicate significant differences between the two groups.

Characteristics	Low RP215	High RP215	*P* value	Statistical methods
*n*	53	47		
Age, median (IQR)	55(50, 60)	58(51, 63)	0.261	Wilcoxon
Gender, *n*(%)			0.563	Chisq test
Male	41(41%)	34(34%)		
Female	12(12%)	13(13%)		
Growth, *n*(%)			0.076	Chisq test
Exogenic type	23(23%)	13(13%)		
Ulcerative type	10(10%)	18(18%)		
Infiltrating type	20(20%)	16(16%)		
Tobacco, *n*(%)			**0.018**	**Chisq test**
No	27(27%)	13(13%)		
Yes	26(26%)	34(34%)		
Alcohol, *n*(%)			0.303	Chisq test
Yes	21(21%)	14(14%)		
No	32(32%)	33(33%)		
T stage, *n*(%)			**0.047**	**Chisq test**
T1	12(12%)	7(7%)		
T2	25(25%)	16(16%)		
T3	7(7%)	4(4%)		
T4	9(9%)	20(20%)		
N stage, *n*(%)			**0.034**	**Yates’s correction**
N0	45(45%)	30(30%)		
N1	4(4%)	5(5%)		
N2	4(4%)	12(12%)		
Metastasis, *n*(%)			**0.003**	**Chisq test**
No	39(39%)	21(21%)		
Yes	14(14%)	26(26%)		
Pathologic grade, *n*(%)			0.397	Yates’s correction
G1	29(29%)	20(20%)		
G2	21(21%)	25(25%)		
G3	3(3%)	2(2%)		
Stage, *n*(%)			0.128	Chisq test
Stage I	11(11%)	6(6%)		
Stage II	18(18%)	10(10%)		
Stage III	8(8%)	6(6%)		
Stage IV	16(16%)	25(25%)		

IQR, interquartile range; Chisq, chi-square

### SIA-IgG promotes the malignant progression of OSCC

**Fig. 2.**
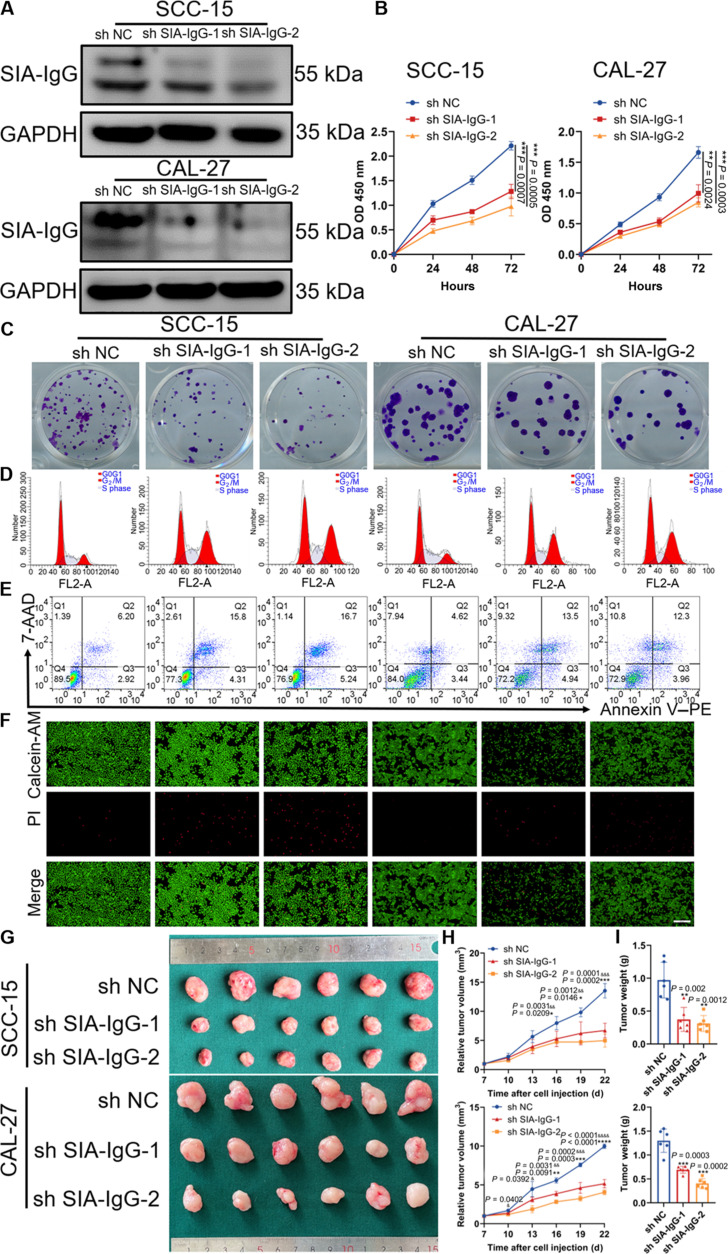
SIA-IgG promotes the malignant progression of OSCC. (A) Western blot was performed to determine SIA-IgG levels within SIA-IgG knockdown SCC-15 and CAL-27 cells. (B) Cell proliferation ability in each group; *n* = 3 per group. (C) Clone images of both cell lines. (D) Cell cycle distribution maps of each group. (E) Apoptosis plots of cells in each group. (F) Calcein-AM/propidium iodide (PI) staining plots of cells from diverse groups (magnification ×10; scale bar, 200 μm). (G) Typical macroscopic images of the tumors in nude mice. (H) Tumor volume of mice injected with SCC-15 (up) and CAL-27 (bottom) cells; *n* = 6 per group. (I) Tumor weight of mice injected with SCC-15 (up) and CAL-27 (bottom) cells; *n* = 6 per group. In (H), * indicates differences between sh NC and sh SIA-IgG-1 groups, and & indicates differences between sh NC and sh SIA-IgG-2 groups. * and &, *P* < 0.05; ** and &&, *P* < 0.01; *** and &&&, *P* < 0.001; and **** and &&&&, *P* < 0.0001 by one-way analysis of variance (ANOVA) with post hoc test. sh NC, short hairpin negative control; GAPDH, glyceraldehyde-3-phosphate dehydrogenase; OD, optical density; 7-AAD, 7-aminoactinomycin D; PE, phycoerythrin.

For exploring SIA-IgG’s possible effect on OSCC, SIA-IgG was knocked down within SCC-15 and CAL-27 cells (Fig. 2A and Fig. S2A to C). As a result, cell proliferation (Fig. 2B) and colony formation ability (Fig. 2C and Fig. S2D) significantly declined in both cell lines following SIA-IgG knockdown. Flow cytometry analysis revealed that SIA-IgG knockdown arrested cell cycle at the G_2_/M phase (Fig. 2D and Fig. S2E) and the cell apoptosis rate increased significantly (Fig. 2E and Fig. S2F). From western blot results, proliferation-related protein proliferating cell nuclear antigen (PCNA) and anti-apoptotic protein Bcl-2 levels declined, while apoptotic proteins Bax and cleaved caspase-3 levels increased after SIA-IgG knockdown (Fig. S2G and H). Calcein-AM/propidium iodide (PI) double staining demonstrated that the PI-labeled dead cell proportion markedly elevated following SIA-IgG knockdown (Fig. 2F and Fig. S2I). According to the above results, SIA-IgG enhanced malignant behavior in OSCC cells in vitro. To further elucidate whether SIA-IgG promoted OSCC progression in vivo, we established an OSCC xenograft model in nude mice by subcutaneously injecting cells from each group. Collectively, the SIA-IgG knockdown group had significantly decreased tumor growth rate and weight relative to those of the control group (Fig. 2G to I). These findings support that SIA-IgG enhances OSCC progression in vitro and in vivo.

### SIA-IgG is critical to OSCC CSC stemness

**Fig. 3.**
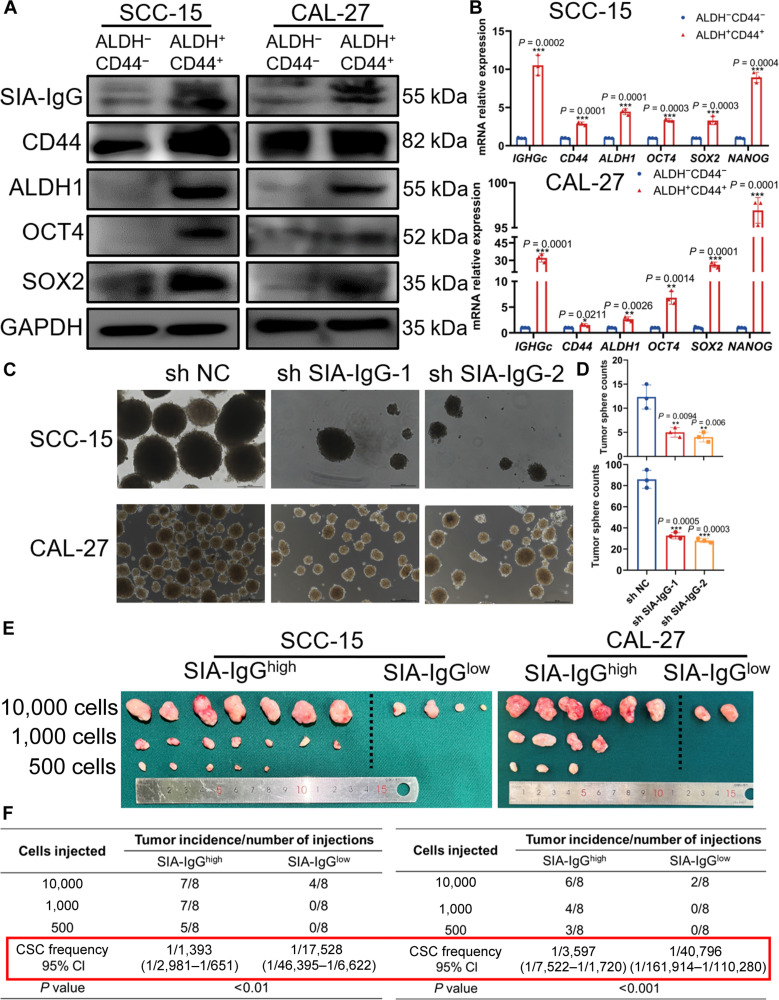
SIA-IgG is critical to the stemness of OSCC cancer stem cells (CSCs). (A and B) Western blot and real-time polymerase chain reaction (RT-PCR) analyses (*n* = 3 per group) of SIA-IgG and CSC marker proteins in CD44^+^/ALDH^+^ and negative cells, respectively. (C and D) Representative tumor sphere images (C) (magnification ×10; scale bar, 250 μm) and statistical analyses (D) of SCC-15 (up) and CAL-27 (bottom) cells, separately; *n* = 3 per group. (E) Typical macroscopic images showing nude mouse tumors. (F) The proportion of CSCs and the corresponding *P* values of diverse groups; *n* = 8 per group. *P < 0.05, **P < 0.01, and ***P < 0.001 by one-way ANOVA or unpaired *t* test as appropriate. ALDH1, aldehyde dehydrogenase 1; OCT4, octamer-binding transcription factor 4; SOX2, SRY-box transcription factor 2‌; mRNA, messenger RNA; CI, confidence interval.

In previous studies, SIA-IgG may be the marker of CSCs in breast and lung cancers [11,29]; however, the relation of SIA-IgG to CSCs in OSCC remains unclear. We first examined SIA-IgG and CSC marker levels within OSCC cells through western blot (Fig. S3A and B), as well as flow cytometry (Fig. S3C and D). The results demonstrated a consistent trend in the expression changes of SIA-IgG and CSC markers across different cell lines. Subsequently, we sorted OSCC CSCs (CD44^+^/ALDH^+^) and non-CSCs (CD44^−^/ALDH^−^) (Fig. S3E). As discovered, relative to those in non-CSC cells, both SIA-IgG and CSC marker levels were significantly up-regulated in CSCs (Fig. 3A and B and Fig. S3F). Thus, the SIA-IgG level was positively related to the tumor stemness of OSCC. Next, we seeded cells into low-attachment plates for suspension tumor sphere culture. Following SIA-IgG knockdown, the tumor-sphere-forming potential of the cells was weakened, with tumor spheres being significantly smaller and fewer in number compared to those in the control group (Fig. 3C and D), suggesting that SIA-IgG promotes the maintenance of stemness in OSCC CSCs in vitro.

To elucidate the in vivo effects of SIA-IgG on OSCC CSCs, we labeled SIA-IgG with Cy3, sorted positive and negative cells from the cell population, and designated them as SIA-IgG^high^ and SIA-IgG^low^, respectively. Cells were collected and injected subcutaneously into mice at densities of 500, 1,000, and 10,000 cells to observe tumor progression (Fig. S4A and B). The results showed that SIA-IgG^high^ cells derived from both cell lines were capable of forming tumors at all 3 densities (the tumor formation rates were 7/8, 7/8, and 5/8 for SCC-15 cells and 6/8, 4/8, and 3/8 for CAL-27 cells). In contrast, SIA-IgG^low^ cells formed tumors only at a density of 10,000 cells, with tumor formation rates of 4/8 and 2/8 (Fig. 3E and Fig. S4C). Moreover, the Extreme Limiting Dilution Analysis (ELDA) software calculation results showed that the proportion of CSCs in SIA-IgG^high^ cells was significantly higher than that in SIA-IgG^low^ cells (Fig. 3F). Immunofluorescence (IF) analysis of the tumor samples revealed extremely weak SIA-IgG expression in the SIA-IgG^low^ group, whereas the SIA-IgG^high^ group exhibited a high positivity rate for SIA-IgG, corresponding to the tumor formation rates mentioned above (Fig. S4D). Collectively, these results indicate that SIA-IgG^high^ cells possess a strong tumor-forming ability with a low cell number in vivo and exhibit self-renewal characteristics typical of CSCs.

**Fig. 4.**
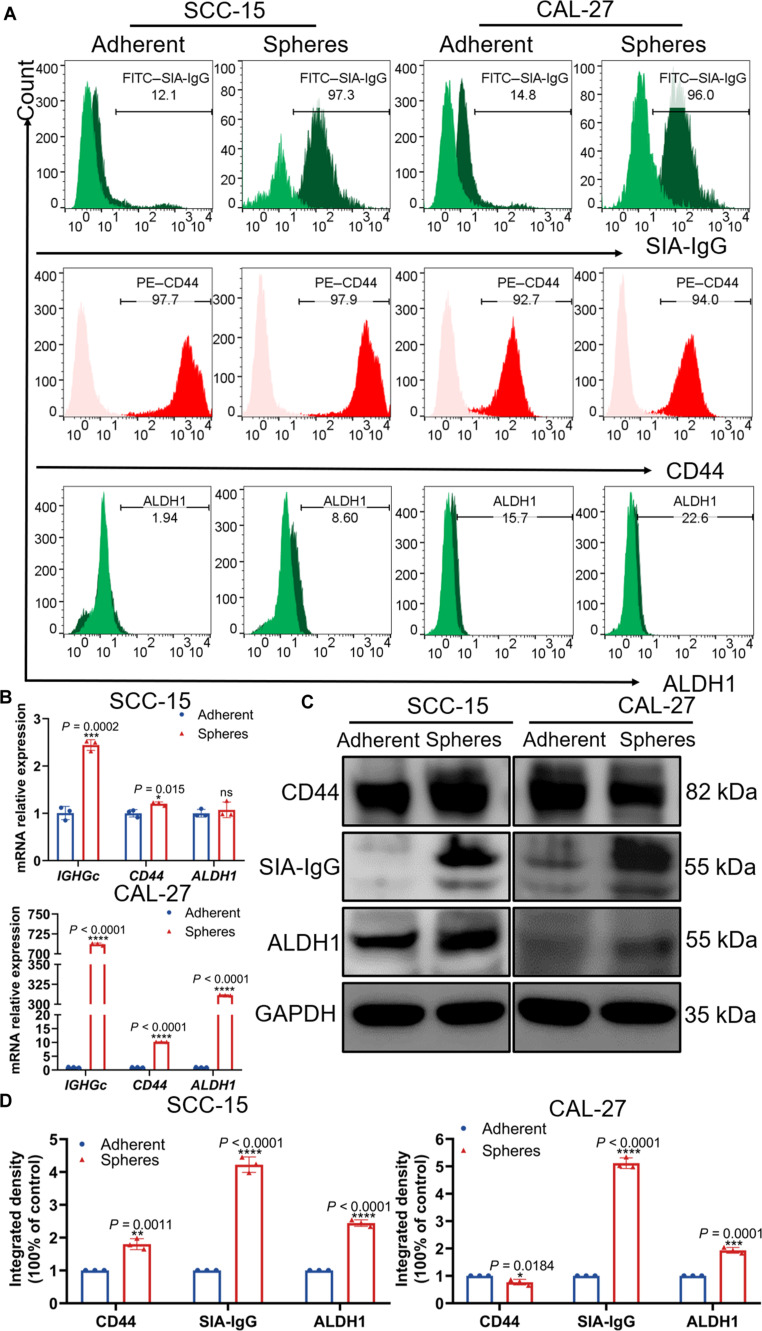
SIA-IgG is a potential biomarker for OSCC CSCs. (A to C) CD44, ALDH1, and SIA-IgG levels within adherent and sphere culture were observed through flow cytometry, RT-PCR (*n* = 3 per group), and western blot, separately.(D) Quantitative analysis of (C); *n* = 3 per group. ns, *P* > 0.05; *P < 0.05; **P < 0.01; ***P < 0.001; and ****P < 0.0001 by unpaired *t* test. FITC, fluorescein isothiocyanate.

As suggested by the aforementioned findings, SIA-IgG may be a surface marker of OSCC CSCs. Therefore, we obtained OSCC CSCs through suspension culture, defined adherent cells as non-CSCs, and then examined the expression of CD44, aldehyde dehydrogenase 1 (ALDH1), and SIA-IgG in the 2 groups. Among the 3 markers, SIA-IgG was the most significantly expressed in the 2 groups (Fig. 4A to C). In summary, in vitro and in vivo analyses revealed SIA-IgG’s crucial effect on OSCC cell stemness. Furthermore, compared with CD44 and ALDH1, SIA-IgG exhibits substantial potential as a marker for OSCC CSCs and can serve as a robust complementary marker to the existing ones at minimum.

### SIA-IgG enhances OSCC malignant progression by activating OXPHOS

**Fig. 5.**
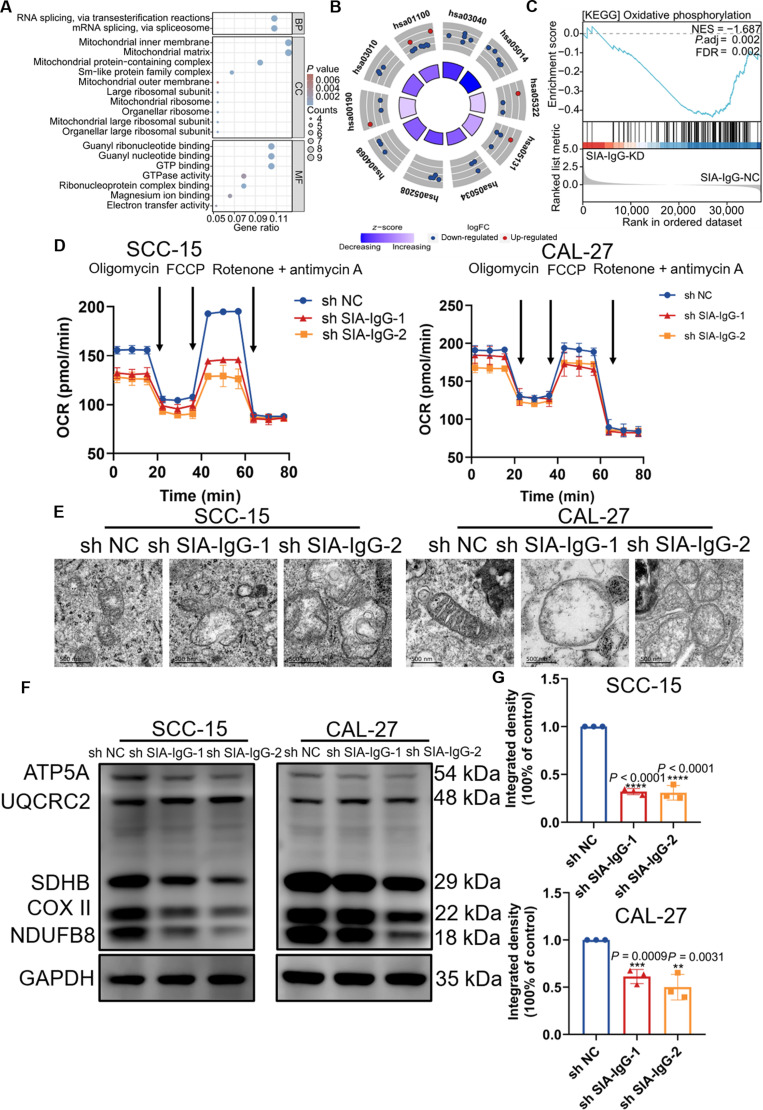
SIA-IgG can activate the oxidative phosphorylation (OXPHOS) pathway in OSCC. (A to C) Gene Ontology (GO), Kyoto Encyclopedia of Genes and Genomes (KEGG), and gene set enrichment analysis (GSEA) enrichment map of differential proteins, respectively. (D) Oxygen consumption rate (OCR) measurement of OSCC cells in each group; *n* = 3 per group. (E) Mitochondrial morphology diagram (magnification ×6,000; scale bar, 500 nm). (F) Western blot analyses of the OXPHOS complex in OSCC cells. (G) Quantitative analysis of (F); *n* = 3 per group. **P < 0.01, ***P < 0.001, and ****P < 0.0001 by one-way ANOVA with post hoc test. GTP, guanosine triphosphate; BP, biological processes; CC, cellular components; MF, molecular functions; FC, fold change; NES, normalized enrichment score; FDR, false discovery rate; KD, knockdown; FCCP, carbonyl cyanide 4-(trifluoromethoxy) phenylhydrazone; ATP5A, ATP synthase F1 subunit alpha; UQCRC2, ubiquinol cytochrome C reductase core protein 2; SDHB, succinate dehydrogenase complex iron sulfur subunit B; COX II, complex IV subunit II; NDUFB8, NADH:ubiquinone oxidoreductase subunit B8.

To elucidate the mechanism by which SIA-IgG facilitates malignant progression in OSCC, we conducted proteomic profiling to identify the differentially expressed proteins following SIA-IgG knockdown (Fig. S5A to E). We carried out enrichment analysis on the 78 differentially expressed proteins. Gene Ontology annotation, comprising biological processes, cellular components, and molecular functions, revealed a strong association with mitochondria (Fig. 5A). From Kyoto Encyclopedia of Genes and Genomes analysis, most differentially expressed proteins were related to metabolism-related pathways (Fig. 5B and Fig. S5F). Additionally, gene set enrichment analysis suggested that OXPHOS was significantly up-regulated in SIA-IgG^high^ samples (Fig. 5C). Subsequently, we confirmed the regulatory role of SIA-IgG in OXPHOS by measuring the oxygen consumption rate (OCR) of OSCC cells using Seahorse XF Analyzer. The results showed that SIA-IgG knockdown reduced the OCR parameters (Fig. 5D and Fig. S5G), including basal respiration, ATP production, proton leak, and maximal respiration. Concurrently, transmission electronic microscopy revealed abnormal mitochondrial morphology in SIA-IgG knockdown cells, characterized by swelling, disappearance of cristae, and vacuolization (Fig. 5E). Furthermore, western blot analysis of the OXPHOS complex expression demonstrated varying degrees of down-regulation following SIA-IgG knockdown (Fig. 5F and G). We further used the OXPHOS inhibitor metformin to co-culture OSCC cells with the short hairpin negative control (sh NC) group and the sh SIA-IgG group, respectively. The preliminary results indicated that metformin and knockdown SIA-IgG had a synergistic effect; compared with metformin alone or knockdown SIA-IgG alone, they could more significantly inhibit the proliferation of OSCC cells (Fig. S5H and I). Collectively, these findings suggest that SIA-IgG promotes the malignant progression of OSCC by influencing OXPHOS-related genes, thereby inducing OXPHOS activation.

### SIA-IgG interacts with VCP to inhibit the degradation of the NDUFB6 protein

**Fig. 6.**
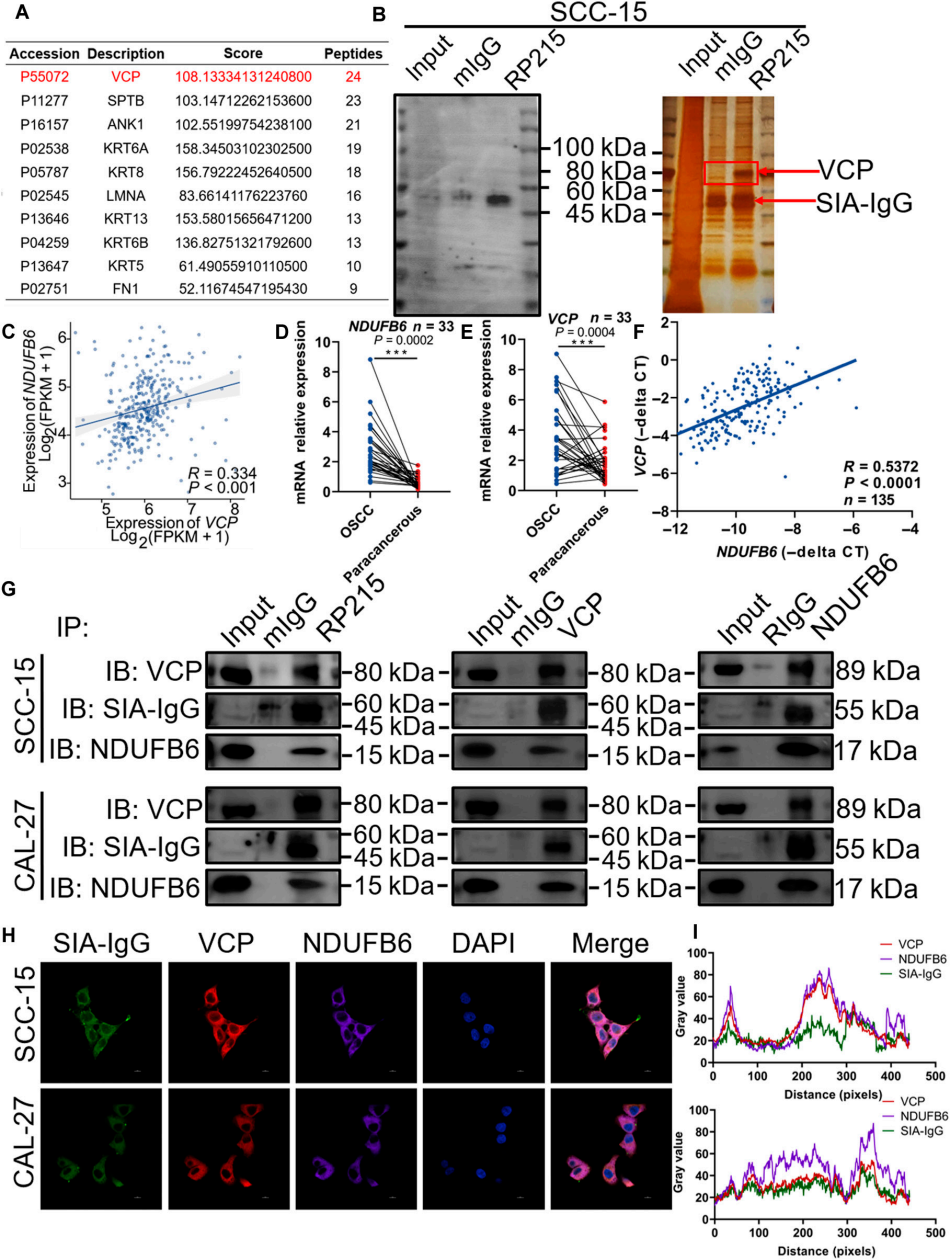
SIA-IgG, valosin-containing protein (VCP), and NADH:ubiquinone oxidoreductase subunit B6 (NDUFB6) interact in OSCC cells. (A) The list of SIA-IgG-binding proteins identified by immunoprecipitation coupled with liquid chromatography/mass spectrometry (LC/MS) analyses. (B) Western blot band verification images (left) after immunoprecipitation using RP215; silver staining proteins immunoprecipitated with SIA-IgG show a specific band at 80 to 100 kDa corresponding to VCP (right). (C) Correlation analysis of *VCP* and *NDUFB6* in the TCGA database; *n* = 330. (D and E) RT-PCR analyses of *NDUFB6* (D) and *VCP* (E) in OSCC and paracancerous tissues; *n* = 33 per group. (F) Correlation analysis of *VCP* and *NDUFB6* in 135 OSCC tissues; *n* = 135. (G) The interaction between SIA-IgG, VCP, and NDUFB6 was demonstrated by co-immunoprecipitation (co-IP) assays. (H) Immunofluorescence (IF) recognizes the co-localization of SIA-IgG, VCP, and NDUFB6 in OSCC cells. SIA-IgG was labeled with TYR-520 (green), VCP with TYR-570 (red), and NDUFB6 with TYR-690 (purple) (magnification ×100; scale bar, 10 μm). (I) Fluorescence distribution maps of SIA-IgG, VCP, and NDUFB6. ***P < 0.001 by paired *t* test and Pearson for correlation analysis. IP, immunoprecipitation; IB, immunoblotting; mIgG, mouse immunoglobulin G; RIgG, rabbit immunoglobulin G; CT, cycle threshold; DAPI, 4′,6-diamidino-2-phenylindole.

For analyzing the molecular mechanism of SIA-IgG in regulating OXPHOS, we identified the binding partners of SIA-IgG through immunoprecipitation–mass spectrometry (IP–MS). The top 10 potential binding proteins are depicted in Fig. 6A. We selected VCP (a specific band at 80 to 100 kDa; Fig. 6B) for subsequent validation, revealing its MS/MS spectrum with the identified amino acid sequence interacting with SIA-IgG marked in red (Fig. S6A and B). Subsequently, TCGA-derived OSCC patients were categorized as a high- or a low-expression group according to the median VCP level. The association between VCP and OXPHOS genes enriched in Fig. 5B was calculated, revealing a significant correlation between VCP and NDUFB6 (Fig. 6C and Fig. S6C). Furthermore, western blotting and real-time polymerase chain reaction (RT-PCR) results demonstrated that both VCP and NDUFB6 expression increased within OSCC samples relative to that in adjacent noncarcinoma samples, with consistent expression trends (Fig. 6D and E and Fig. S6D). The correlation between *VCP* and *NDUFB6* messenger RNA levels was analyzed in 135 OSCC samples, which showed a significant correlation, further validating the database analysis results (Fig. 6F).

**Fig. 7.**
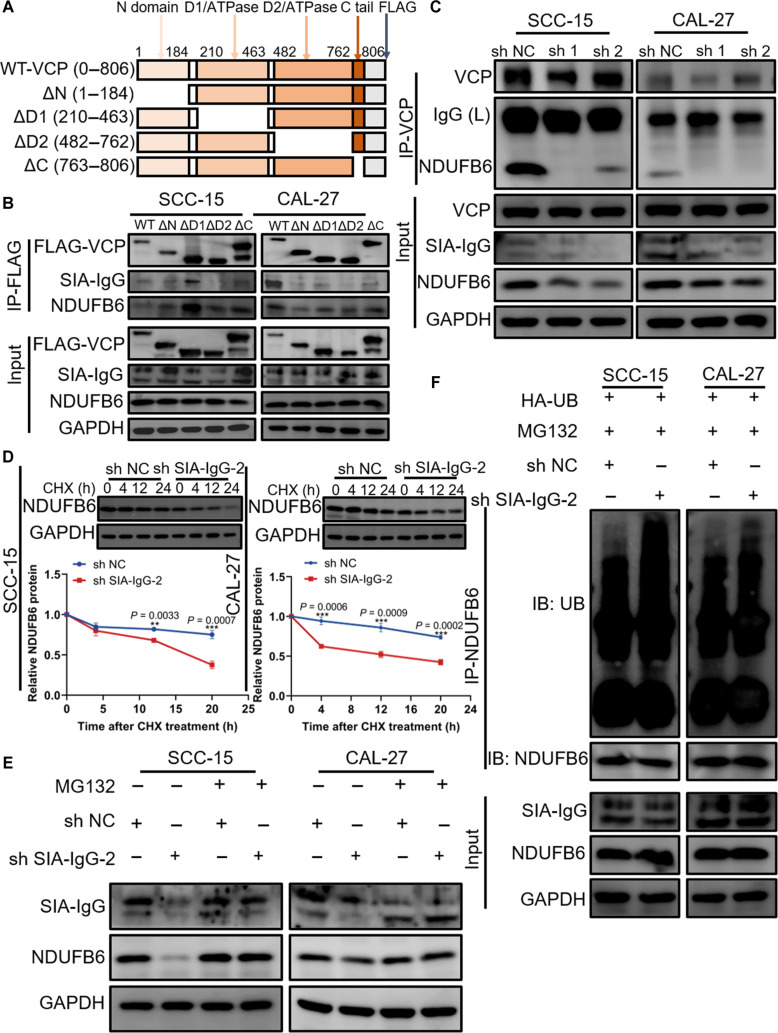
SIA-IgG interacts with VCP to inhibit the degradation of the NDUFB6 protein. (A) Schematics of the structural domains of VCP: The wild-type (WT) VCP plasmid contains 806 amino acids and consists of 4 domains in total. The blank regions represent the deleted region. (B) Co-IP assays were employed to detect the interactions between SIA-IgG and NDUFB6 with VCP-truncation mutants (WT, ΔN, ΔD1, ΔD2, and ΔC). (C) OSCC cells were transfected either with or without SIA-IgG knockdown plasmids, IP was performed using anti-VCP antibodies, and the NDUFB6 level was evaluated through western blot. (D) After treating each group of cells with cycloheximide (CHX; 10 μg/ml) for 0, 4, 12, and 24 h, the NDUFB6 protein level was detected through western blot and quantitatively analyzed; *n* = 3 per group. (E) After treating the control group and the SIA-IgG knockdown group with or without MG132 (10 μM) for 12 h, SIA-IgG and NDUFB6 protein levels were examined through western blot. (F) Co-IP was performed to detect ubiquitin bound to NDUFB6 following SIA-IgG knockdown. **P < 0.01 and ***P < 0.001 by unpaired *t* test. HA-UB, hemagglutinin-ubiquitin; UB, ubiquitin.

Co-immunoprecipitation (co-IP; Fig. 6G) and IF analyses (Fig. 6H and I) confirmed the interactions among SIA-IgG, VCP, and NDUFB6, indicating their co-localization in the cytoplasmic region of OSCC cells. To map the interaction domains in detail, we generated 4 truncation mutants of VCP: ΔN (deletion of amino acids 1 to 184), ΔD1 (deletion of amino acids 210 to 463), ΔD2 (deletion of amino acids 482 to 762), and ΔC (deletion of amino acids 763 to 806) (Fig. 7A). Through co-IP analysis, we found that SIA-IgG binds to the D2 domain of VCP, whereas NDUFB6 can bind to each domain of VCP (Fig. 7B).

Given that VCP is a molecular chaperone that inhibits the degradation of multiple downstream molecules, we hypothesized that SIA-IgG may enhance VCP’s ability to capture and stabilize its downstream molecule NDUFB6 by reducing its degradation. To verify this hypothesis, co-IP experiments were performed on OSCC cells with or without SIA-IgG knockdown. The results showed that the binding between VCP and NDUFB6 was weakened due to SIA-IgG knockdown, indicating that VCP regulated by SIA-IgG has a higher affinity for NDUFB6 and can stabilize its expression (Fig. 7C). Subsequently, the cells were treated with cycloheximide, and the results demonstrated that the degradation rate of the NDUFB6 protein was significantly accelerated after SIA-IgG knockdown (Fig. 7D). Moreover, treatment with the proteasome inhibitor MG132 rescued the decreased expression of NDUFB6 caused by SIA-IgG knockdown (Fig. 7E), and SIA-IgG attenuated the ubiquitination of NDUFB6 (Fig. 7F). In summary, these findings suggest that SIA-IgG binds to the D2 domain of VCP to inhibit the ubiquitination–proteasome-pathway-mediated degradation of NDUFB6, thereby stabilizing its protein expression.

### SIA-IgG promotes OSCC progression through enhancing NDUFB6 expression and activating OXPHOS

**Fig. 8.**
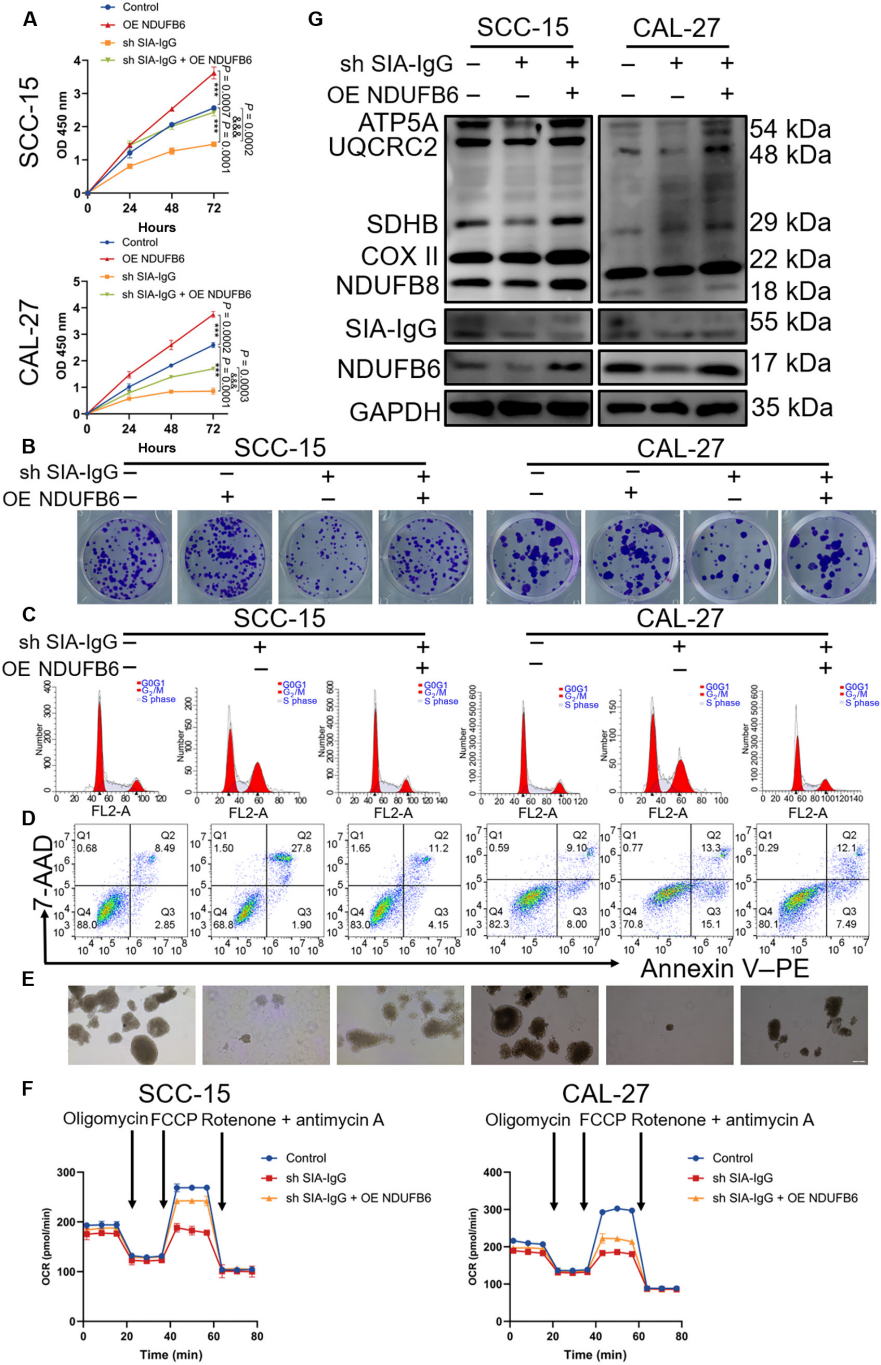
SIA-IgG can enhance the expression of NDUFB6 and thereby activate OXPHOS in OSCC. Detection of cell proliferation ability (A), clone images (B), cell cycle distribution maps (C), apoptosis plots (D), tumor sphere images (magnification ×10; scale bar, 200 μm) (E), the measurement of OCR (F), and the detection of OXPHOS-related proteins in each group (G); *n* = 3 per group. In (A), * indicates difference of the control group versus the SIA-IgG knockdown or NDUFB6 overexpression group, and & indicates difference of the SIA-IgG knockdown group versus the SIA-IgG knockdown + NDUFB6 overexpression group. *** and &&&, *P* < 0.001 by one-way ANOVA with post hoc test. OE, overexpression.

To further substantiate that SIA-IgG influences OXPHOS by modulating NDUFB6 expression levels, thereby promoting OSCC malignant progression, we carried out diverse experiments where an NDUFB6-overexpressing plasmid was introduced into SIA-IgG knockdown cells. The results indicated that simple overexpression of NDUFB6 could significantly promote the proliferation of OSCC cells. The proliferation inhibition produced after SIA-IgG knockdown could also be restored due to the overexpression of NDUFB6, but it was far from the level when NDUFB6 was overexpressed alone (Fig. 8A and B and Fig. S7A). It can be inferred from this that NDUFB6 plays an important role in SIA-IgG promoting the malignant progression of OSCC. Furthermore, our subsequent experimental results also confirmed that NDUFB6 overexpression reversed cell cycle arrest (Fig. 8C and Fig. S7B) and increased apoptosis (Fig. 8D and Fig. S7C) and elevated cell death (Fig. S7E and F) caused by SIA-IgG depletion. Meanwhile, the growth potential of tumor spheres weakened by SIA-IgG knockdown was also restored (Fig. 8E and Fig. S7D); various parameters of OCR and OXPHOS-related proteins were also restored to varying degrees (Fig. 8F and G and Fig. S7G and H). Collectively, in OSCC, SIA-IgG activates the OXPHOS pathway by increasing NDUFB6 protein expression, thereby promoting malignant progression.

### VCP is essential for SIA-IgG-induced promotion of NDUFB6 expression, activation of OXPHOS, and malignant progression in OSCC

**Fig. 9.**
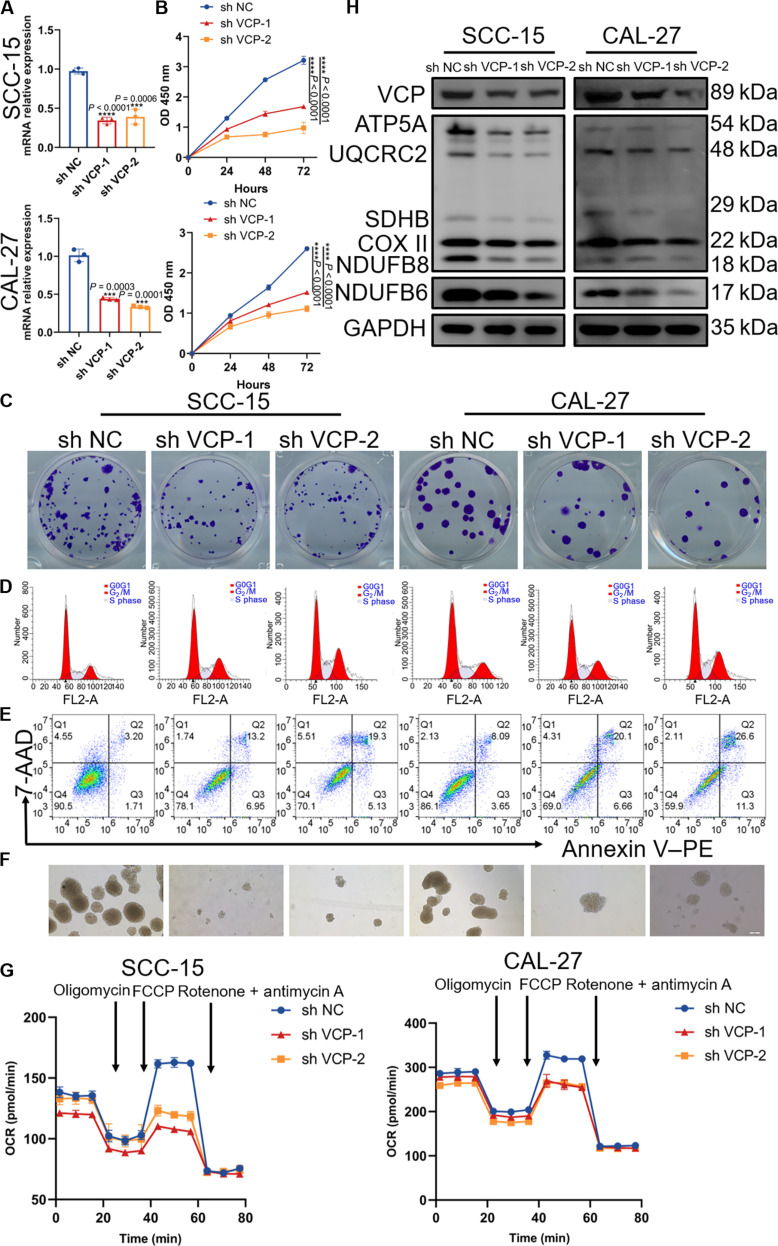
VCP promotes the malignant progression of OSCC. (A) SCC-15 and CAL-27 cells underwent transfection using 2 anti-VCP short hairpin RNA (shRNA), and the knockdown effects were detected by RT-PCR analysis; *n* = 3 per group. (B to H) Detection of cell proliferation ability (B), clone images (C), cell cycle distribution maps (D), apoptosis plots (E), tumor sphere images (magnification ×10; scale bar, 200 μm) (F), the measurement of OCR (G), and the detection of OXPHOS-related and VCP proteins in each group (H); *n* = 3 per group. In (A) and (B), * indicates comparison of the control group versus the VCP knockdown groups. ***P < 0.001 and ****P < 0.0001 by one-way ANOVA with post hoc test.

**Fig. 10.**
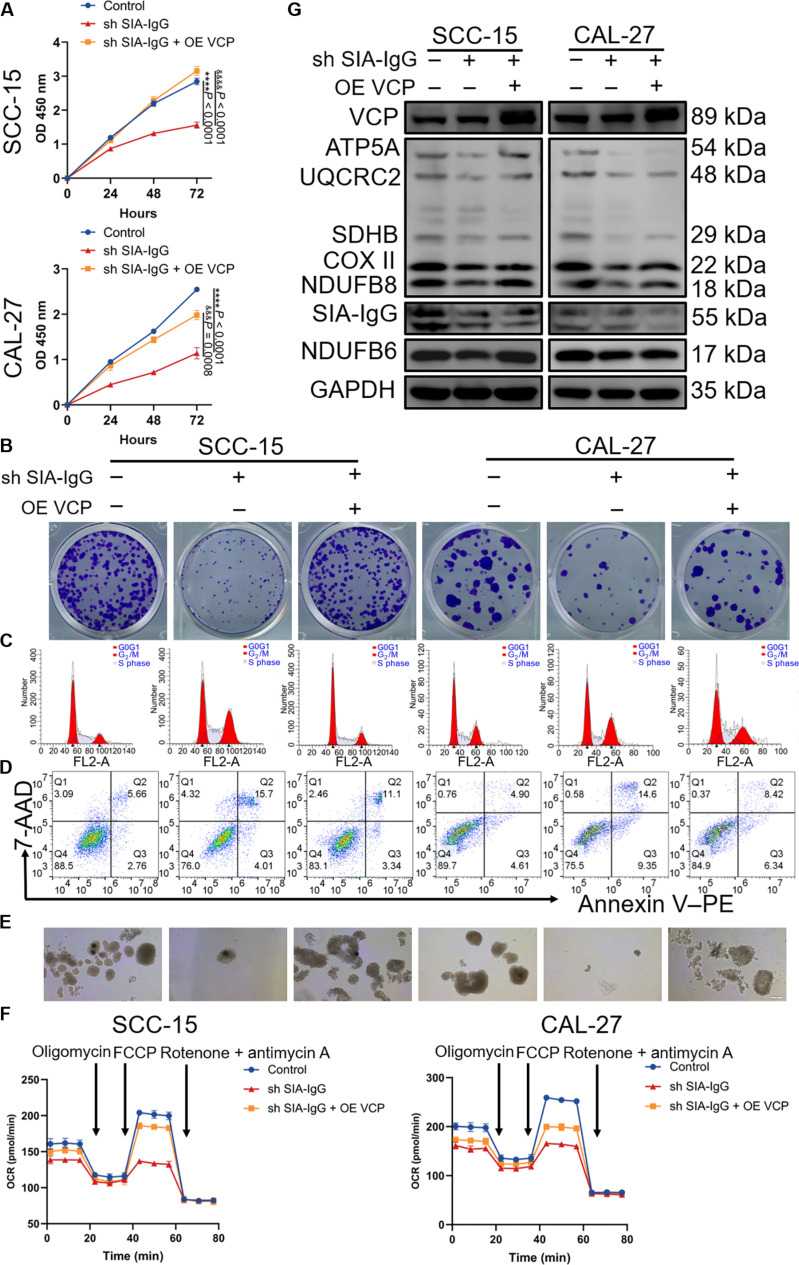
VCP overexpression suppresses the promalignant progression effect of SIA-IgG. Detection of cell proliferation ability (A), clone images (B), cell cycle distribution maps (C), apoptosis plots (D), tumor sphere images (magnification ×10; scale bar, 200 μm) (E), the measurement of OCR (F), and the detection of OXPHOS-related and VCP proteins in each group (G); *n* = 3 per group. In (A), * indicates difference of the control group versus the SIA-IgG knockdown group, and & indicates difference of the SIA-IgG knockdown group versus the SIA-IgG knockdown + VCP overexpression group. &&&, *P* < 0.001, and **** and &&&&, *P* < 0.0001 by one-way ANOVA with post hoc test.

VCP’s effect on OSCC malignant progression and its relationship with OXPHOS is still unclear. According to our findings, VCP knockdown (Fig. 9A and H) inhibited cell proliferation (Fig. 9B and C and Fig. S8A), induced cell cycle arrest at the G_2_/M phase (Fig. 9D and Fig. S8B), increased apoptosis (Fig. 9E and Fig. S8C), and elevated cell death (Fig. S8E and F). Additionally, VCP knockdown suppressed the tumor-sphere-forming potential (Fig. 9F and Fig. S8D) and OXPHOS activity (Fig. 9G and H and Fig. S8G and H). We then verified whether VCP is essential for SIA-IgG-induced malignant progression in OSCC by using a VCP-overexpressing plasmid. The results demonstrated that VCP overexpression mitigated the suppressed OSCC cell proliferation resulting from SIA-IgG knockdown (Fig. 10A and B and Fig. S9A) and reversed the cell cycle arrest (Fig. 10C and Fig. S9B), the increased apoptosis (Fig. 10D and Fig. S9C), and the elevated cell death (Fig. S9E and F). It also restored tumor-sphere-forming potential (Fig. 10E and Fig. S9D), which was diminished by SIA-IgG knockdown, along with varying degrees of recovery in OCR parameters (Fig. 10F and Fig. S9G) and OXPHOS-related proteins (Fig. 10G and Fig. S9H). Such results highlight the key effect of VCP on SIA-IgG-induced promotion of NDUFB6 expression, activation of OXPHOS, and malignant progression in OSCC.

### The SIA-IgG-specific antibody RP215 exhibits clear antitumor therapeutic effects

**Fig. 11.**
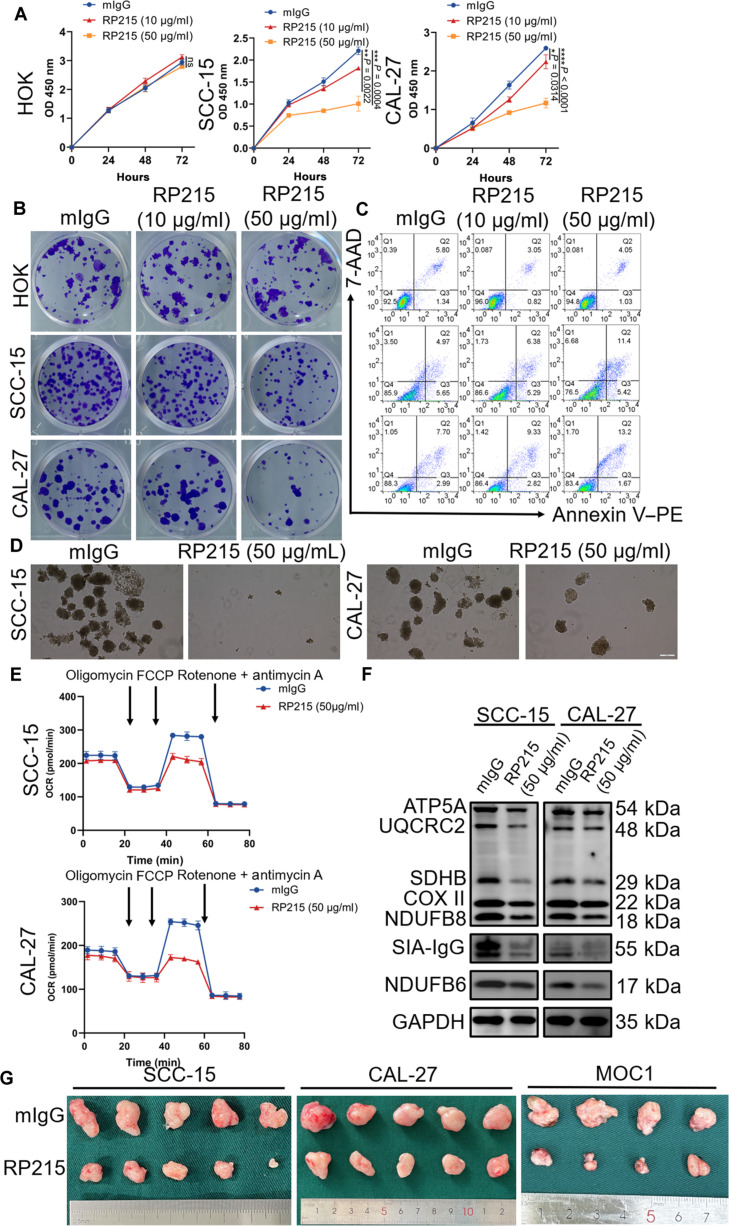
RP215 inhibits the malignant progression of OSCC. (A) 10 and 50 μg/ml RP215 and 50 μg/ml mIgG were added to detect their effects on the cell proliferation ability of human oral keratinocyte (HOK), SCC-15, and CAL-27 cells; *n* = 3 per group. (B to F) Clone images, apoptosis plots, tumor sphere images (magnification ×10; scale bar, 200 μm), the measurement of OCR, and the detection of OXPHOS-related proteins in each group, respectively; *n* = 3 per group. (G) Representative macroscopic images of the tumors formed of SCC-15 (*n* = 5 per group), CAL-27 (*n* = 5 per group), and MOC1 (*n* = 4 per group), respectively. * indicates comparison between the RP215 and mIgG groups. ns, *P* > 0.05; *P < 0.05; **P < 0.01; ***P < 0.001; and ****P < 0.0001 by one-way ANOVA with post hoc test.

As suggested by the aforementioned results, SIA-IgG is a novel anti-OSCC target. We carried out in vitro and in vivo experiments using the SIA-IgG-specific antibody RP215. Compared with control mouse IgG (mIgG), RP215 dramatically suppressed OSCC cell proliferation and promoted apoptosis but had no obvious effect on human oral keratinocyte (HOK) cells (Fig. 11A to C and Fig. S10A and B). It also markedly suppressed tumor sphere formation (Fig. 11D and Fig. S10C) and OXPHOS activity (Fig. 11E and F and Fig. S10D and E). To determine in vivo effects, we established xenograft tumor and syngeneic tumor models and treated the mice with 5 mg/kg RP215 or mIgG via tail vein injection. From these findings, relative to the mIgG group, RP215 apparently suppressed OSCC growth and decreased tumor weight (Fig. 11G and Fig. S10F and G). Protein analysis of mouse tumor tissues revealed that SIA-IgG, OXPHOS-related protein levels, and NDUFB6 significantly declined in the RP215 treatment group (Fig. S10H). Hematoxylin–eosin (HE) staining showed the absence of toxic effect on mouse internal organs (Fig. S11A). For tumors formed by MOC1 cells, HE staining and IHC were employed to evaluate tumor cell morphology and the blocking effect of SIA-IgG. The results revealed that in the RP215 group, the density of tumor cells was reduced and the expression of SIA-IgG was significantly diminished (Fig. S11B and C). According to blood routine and biochemical tests, RP215 did not affect blood cell indices, and liver and kidney function indices fluctuated within the normal range, suggesting no damage to mouse organ function (Fig. S11D). In conclusion, the SIA-IgG-targeting antibody RP215 can effectively and safely block SIA-IgG-induced suppression of NDUFB6 mechanism in vivo, thereby inhibiting tumor growth.

## Discussion

Since its discovery, SIA-IgG has been extensively validated by multiple research groups, with accumulated evidence demonstrating its overexpression in various malignant tumors and its critical role in tumor progression [8–11]. Notably, our previous studies confirmed elevated SIA-IgG levels in both OSCC [15] and salivary adenoid cystic carcinoma [30,31], where it was significantly associated with metastasis, recurrence, and poor prognosis [30,31]. Therefore, the present study conducted a more comprehensive exploration of the expression pattern of SIA-IgG in OSCC via expanding the patient cohort. Our results demonstrated for the first time that overexpression of SIA-IgG in OSCC patients was closely related to poor OS. Currently, prognostic indicators for OSCC patients, such as the widely used TNM staging [32], emerging indicators lymph node ratio (LNR) [33], and log odd of positive lymph nodes (LODDS) [34], all exhibit inherent limitations. Therefore, identifying more robust prognostic indicators to assist in clinical decision-making remains a challenging topic. Our analysis integrating SIA-IgG IHC scores with the clinicopathological characteristics of OSCC patients revealed that SIA-IgG outperformed TNM staging in prognostic prediction and emerged as an independent prognostic factor for OSCC patients. Regrettably, due to the insufficient lymph node data, we failed to compare SIA-IgG IHC scores with the LNR and LODDS indicators. Nevertheless, SIA-IgG still demonstrated strong potential as a novel prognostic biomarker for OSCC, warranting further validation in larger, well-annotated cohorts.

CSCs are characterized by their self-renewal capability through asymmetric division, generating one stem-like daughter cell and one differentiated progeny [35]. This unique property of CSCs contributes to sustained tumor growth, therapy resistance, and tumor recurrence [36], making CSC identification and targeted therapeutic strategies critical for OSCC treatment. However, the lack of reliable OSCC CSC markers remains a major challenge. Currently, aldehyde dehydrogenase (ALDH) combined with CD44 represents one of the most widely used approaches for OSCC CSC identification. However, this strategy still has limitations. On one hand, CD44 is highly expressed in both OSCC tissues and normal oral epithelium, impairing its ability to distinguish between malignant and benign tissues [37]. On the other hand, ALDH1, an intracellular enzyme, presents technical challenges in detection and increases assay costs [38]. Our study is the first to demonstrate that SIA-IgG positively correlates with stemness maintenance in OSCC CSCs. In vitro, SIA-IgG promoted tumor sphere formation. Moreover, in vivo, SIA-IgG^high^ OSCC cells exhibited tumorigenic potential, with as few as a small number of cells, indicating that SIA-IgG could serve as a promising OSCC CSCs surface marker. Furthermore, to evaluate its marker potential, we systematically compared the enrichment of SIA-IgG with CD44 and ALDH1 in tumor sphere-enriched CSCs [39]. First, tumor spheres showed significant up-regulation of multiple stem cell markers compared with adherent tumor cells. We then examined the expression levels of CD44, ALDH1, and SIA-IgG in tumor spheres and adherent cells. CD44 showed a positivity rate exceeding 90% in both cases, consistent with prior findings. ALDH1 showed a 4-fold higher expression in CSCs than in adherent cells, whereas SIA-IgG exhibited an 8-fold greater expression in CSCs. These findings suggest that SIA-IgG may serve as a potential OSCC CSC marker for OSCC. However, future studies should explore combinatorial marker strategies incorporating SIA-IgG to isolate purer CSC populations and improve clinical applicability.

With the continuous deepening of basic research on tumors and the continuous improvement of related genetic detection technologies, multi-omic analysis based on gene expression data provides an efficient and convenient means to explore the potential pathogenesis of diseases [40,41]. We also found through proteomic profiling that SIA-IgG is closely related to the OXPHOS pathway in OSCC. OXPHOS is a pivotal regulator of cancer malignancy. It has been reported that cholangiocarcinoma CSCs overexpressed the master regulator of mitochondrial biogenesis, which relied on OXPHOS to develop a more effective respiratory phenotype. Clinically, this metabolic adaptation correlated with poorer outcomes, as patients exhibiting a high OXPHOS activity demonstrated significantly reduced OS and earlier recurrence compared to their low-OXPHOS counterparts [42]. Similarly, in glioblastoma, CSCs were also highly dependent on OXPHOS, and the activation of OXPHOS could lead to treatment failure [43]. Additionally, elevated OXPHOS activity strongly predicts an unfavorable prognosis in OSCC [44]. These studies fully demonstrate that targeting OXPHOS may inhibit the malignant progression of tumors. Our study provides mechanistic insights into OXPHOS regulation in OSCC, revealing that SIA-IgG sustains OXPHOS activation via a novel VCP–NDUFB6 axis. Specifically, SIA-IgG interacts with VCP to stabilize NDUFB6 by inhibiting its proteasomal degradation, thereby activating OXPHOS. These findings reveal the crucial role of SIA-IgG in orchestrating OXPHOS-driven malignancy in OSCC and highlight its potential as a therapeutic target.

**Fig. 12.**
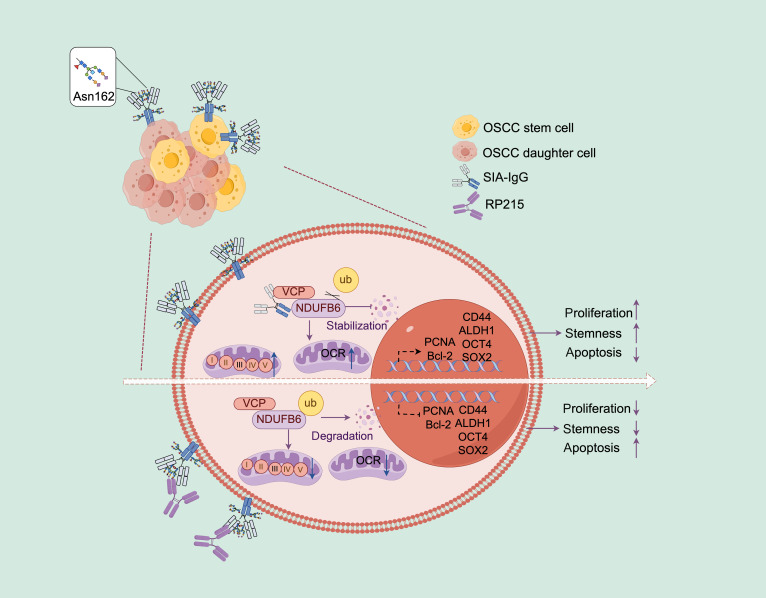
Working model of SIA-IgG on the promotion of OSCC malignant progression. The schematic diagram of the graphical abstract was drawn by Figdraw. PCNA, proliferating cell nuclear antigen; ub, ubiquitin.

VCP serves as a critical oncogenic regulator that regulates diverse cellular processes through its interaction with multiple substrates. Its most well-characterized function involves mediating ubiquitin–proteasome-pathway-dependent protein degradation [25]. It has been reported that depletion of VCP leads to enhanced ubiquitination levels of different protein subsets, such as K6-linked ubiquitination dependent on the ubiquitin E3 ligase HUWE1 [26]. Pu et al. [45] found that in liver cancer, VCP reduces the degradation of HMGB1 via the ubiquitin–proteasome pathway, thereby contributing to the malignant progression of liver cancer. In our study, we identified VCP as a potential binding partner of SIA-IgG using IP–MS analysis. Subsequently, we demonstrated that the binding of SIA-IgG and VCP could enhance the interaction between VCP and NDUFB6, inhibit the ubiquitin–proteasome-mediated degradation of NDUFB6, and eventually activate OXPHOS. Furthermore, knockdown of VCP also led to reduced NDUFB6 expression, indicating a positive correlation between VCP expression and NDUFB6 levels. More importantly, we observed that VCP overexpression attenuated SIA-IgG-driven malignant progression in OSCC, suggesting that SIA-IgG requires VCP to regulate OSCC malignancy (Fig. 12). Our findings align with those of previous research and expand the known substrate repertoire of VCP. However, further in-depth studies are needed to elucidate this mechanism, such as identifying the specific ubiquitin chains or enzymes involved in protein degradation, to provide a more comprehensive understanding of the related mechanisms in OSCC.

## Conclusion

Collectively, this work illustrates the clinical significance and biological mechanisms of SIA-IgG overexpression within OSCC. Mechanistically, SIA-IgG enhances the interaction between VCP and NDUFB6, thereby inhibiting the degradation of NDUFB6, stabilizing its protein expression levels, and activating OXPHOS to promote the malignant progression of OSCC. In addition, blocking SIA-IgG with RP215 antibody may serve as an effective strategy to suppress OSCC progression. Therefore, our results provide a new theoretical foundation for SIA-IgG’s effect on promoting OSCC malignant progression and offer a possible therapeutic strategy targeting SIA-IgG for OSCC therapy.

## Materials and Methods

### Tissues

Tissues were obtained in OSCC cases who underwent radical tumor resection without chemotherapy or radiotherapy before surgery at Peking University School and Hospital of Stomatology (Beijing, China). The samples included OSCC and adjacent nontumor tissues. Corresponding clinical data were collected concurrently, followed by a 3-year follow-up period. The study protocol gained approval from the Biomedical Ethics Committee of Peking University Stomatological Hospital. Every patient provided written informed consent.

### Bioinformatics analysis

Transcriptomic sequencing (RNA sequencing) data and clinical information of OSCC patients were obtained from the TCGA database (https://portal.gdc.cancer.gov/). The clinical correlation between RP215 scores and OSCC patients, the efficacy of diagnosing OSCC, the OS rate, and the identification of independent prognostic factor were analyzed respectively using the “stats”, “pROC”, “survival”, and “rms” packages of the R software. Immunohistochemical staining results of human normal mucosa and OSCC samples were acquired from the Human Protein Atlas database (https://www.proteinatlas.org/).

### Cell lines and cell transfection

The OSCC cell lines employed in this study included WSU-HN6, SCC-9, SCC-15, SCC-25, CAL-27, and MOC1 (murine OSCC cell line), with HOKs serving as nonmalignant control. The WSU-HN6 and HOK cells were provided by Shanghai Ninth People’s Hospital, while the SCC-9, SCC-15, SCC-25, CAL-27, and MOC1 cell lines were provided by the American Type Culture Collection.

All cells underwent culture within complete medium that included 10% fetal bovine serum (Procell, China) as well as 1% penicillin–streptomycin, before incubation under 5% CO_2_ and 37 °C conditions. HOK cells received culture within RPMI 1640 complete medium. WSU-HN6 and CAL-27 cells underwent culture within Dulbecco’s modified Eagle medium (DMEM) complete medium, and SCC-9, SCC-15, and SCC-25 cells, within DMEM/F-12 that contained 400 ng/ml hydrocortisone as well as 1 mM sodium pyruvate, whereas MOC1 cells were cultured in a mixed medium (Iscove’s modified Dulbecco’s medium:DMEM/F-10:DMEM/F-12 = 4:1:1) supplemented with 5 μg/ml insulin (MedChemExpress, USA), 5 ng/ml epidermal growth factor (Sino Biological Inc, China), and 40 ng/ml hydrocortisone (Solarbio, China).

Transfection of short hairpin RNAs (shRNAs) and overexpression plasmids was performed using Lipofectamine 8000 (Beyotime, China), following specific instructions. RT-PCR or western blotting was performed to confirm transfection efficiency. The SIA-IgG shRNA, VCP shRNA, VCP overexpression plasmid, and NDUFB6 overexpression plasmid were purchased from GeneChem (China). Table 2 displays the shRNA sequences utilized in the present work.

**Table 2.** ShRNA sequences

shRNA	Sequence
sh SIA-IgG-1	GGTGGACAAGACAGTTGAG
sh SIA-IgG-2	AGTGCAAGGTCTCCAACAA
sh VCP-1	CCTAGCCCTTATTGCATTGTT
sh VCP-2	CCTGATGTGAAGTACGGCAAA

### Cell cycle and cell apoptosis analysis

Cells were inoculated into 6-well culture dishes (3 × 10^5^ cells/well). In cell cycle analysis, cells were harvested with EDTA-free trypsin, immersed within 70% ethanol overnight at 4 °C, rinsed by phosphate-buffered saline (PBS) twice, and incubated with PI staining reagent that contained RNase A at 37 °C for a 30-min duration according to the cell cycle and apoptosis assay kit instructions (Beyotime, China). To analyze apoptosis, cells underwent 15 min of dual staining using phycoerythrin (PE)-conjugated annexin V and 7-aminoactinomycin D (7-AAD) with Annexin V-PE/7-AAD Apoptosis Assay Kit (Solarbio, China). Samples were filtered through a 300-μm mesh and subjected to flow cytometry analysis using Fortessa (BD Biosciences, USA).

### Cell Counting Kit-8 assay, colony formation assay, and calcein-AM/PI staining

Cell Counting Kit-8 assay (Beyotime, China) was performed for evaluating cell viability following specific protocols. In brief, cells underwent inoculation in 96-well culture plates at 2,000 cells/well with 6 replicates per group. After overnight culture, the plates were subjected to 2 h of probing using 10 μl of Cell Counting Kit-8 reagent within fresh medium (100 μl) at 37 °C. An ELx808 microplate reader (BioTek, USA) was employed for measuring the optical density at 450 nm (OD_450_). The following formula was employed for calculating cell viability: relative change value of OD_450_ = OD in experimental wells − OD in blank wells.

To analyze cell colony formation, we seeded cells (200/well) into 12-well plates before 7 d of culture with medium replenishment at 48-h intervals. After 20 min of fixation using 4% paraformaldehyde, the colonies received additional 20 min of staining using 0.5% crystal violet.

In calcein-AM/PI staining, cells at 80% confluency were incubated for a 30-min duration with calcein-AM/PI cocktail (1 μl of calcein-AM, 1 ml of buffer, and 1 μl of PI) at 37 °C. A fluorescence microscope (Nikon, Japan) was utilized for imaging.

### Western blot

The western blot assay protocol followed that used in our earlier study [46]. In summary, total proteins were isolated in cell lines and OSCC samples with radioimmunoprecipitation assay (RIPA) lysis buffer (Solarbio, China). We utilized BCA Protein Assay Kit (Beyotime, China) for analyzing protein content, following specific instructions. Protein aliquots (20 μg/lane) were subjected to 4% to 20% sodium dodecyl sulfate–polyacrylamide gel electrophoresis (SDS-PAGE) for separation before transfer to polyvinylidene fluoride membranes. After 1.5 h of blockage with 5% bovine serum albumin (Hengyuan, China), membranes underwent overnight incubation at 4 °C with primary antibodies, including glyceraldehyde-3-phosphate dehydrogenase (GAPDH; 1:20,000), ALDH1 (1:1,000), cleaved caspase-3 (1:1,000), and FLAG (1:5,000) (Proteintech, China); SIA-IgG (purified by our laboratory, 1:1,000); PCNA (1:1,000) and CD44 (1:1,000) (FineTest, China); SRY-box transcription factor 2‌ (SOX2; 1:500) and octamer-binding transcription factor 4 (OCT4; 1:500) (Biodragon, China); Bcl-2 associated X protein (Bax; 1:1,000; HUABIO, China) and Bcl-2 (1:1,000; Wanlei, China); VCP (1:1,000) and hemagglutinin (1:5,000; Beyotime, China); NDUFB6 (1:1,000; Novusbio, USA); and Total OXPHOS (1:1,000; Abcam, UK). Then, membranes were further probed using horseradish peroxidase (HRP)-conjugated secondary antibodies for an additional 1 h. Super-Enhanced Chemiluminescence Detection Kit (Beyotime, China) was applied in detecting target proteins. e-BLOT Touch Imager was used to display the protein bands and analyze their gray values.

### RT-PCR analysis

TRIzol reagent was adopted for isolating total RNA following specific protocols. Subsequently, complementary DNA (cDNA) was prepared using the cDNA Reverse Transcription Kit (Takara, Japan). PCR was performed with 2× Universal SYBR Green Fast qPCR Mix (ABclonal, China). RT-PCR was carried out with ABI 7500 Real-Time PCR System (Applied Biosystems, USA), with GAPDH being the housekeeping gene for normalization. Relative quantification of the target gene was performed through 2^−ΔΔCt^. The oligonucleotide primers were purchased from Sangon Biotech (Table 3).

**Table 3.** Primers used in RT-PCR

Primer	Sequence
*GAPDH*	F: GCACCGTCAAGGCTGAGAAC R: TGGTGAAGACGCCAGTGGA
*IGHGc*	F: ACTACAAGACCACGCCTCC R: CGTCGCACTCATTTACCC
*CD44*	F: CAGCTCATACCAGCCATCCA R: GCTTGATGACCTCGTCCCAT
*ALDH1*	F: ATCAAAGAAGCTGCCGGGAA R: GCATTGTCCAAGTCGGCATC
*VCP*	F: AGCCGCGCAGGTTCAAAAG R: GTGTCATCAATGGGCAGCAC
*NDUFB6*	F: TGAGAAGGCGATGGCTGAAG R: GCCATATGGTTTTTCCATTTTCCT

### Co-IP, MS, and proteomic analysis

Co-IP was conducted using Immunoprecipitation Kit (Beyotime, China) following the manufacturer’s protocol with minor adjustments. Briefly, cells were lysed by RIPA buffer containing protease-inhibitor cocktail and later incubated using specific primary antibodies at 4 °C overnight. The protein–antibody complex was then probed using protein A/G beads at 4 °C for a 2-h duration. Finally, the immunoprecipitated complexes were subjected to 10 min of elution using SDS-PAGE loading buffer at 95 °C before western blot assay.

MS analysis was carried out to identify the interacting protein partners of SIA-IgG. The immunoprecipitated complexes were resolved by SDS-PAGE and visualized using Rapid Silver Staining Kit (Beyotime, China). Protein bands of the corresponding molecular weights were excised and processed by sequential destaining, tryptic in-gel digestion, and peptide extraction. Purified tryptic peptides were analyzed using Orbitrap Fusion Lumos Mass Spectrometer (Thermo, USA).

For investigating the molecular mechanism by which SIA-IgG regulated OSCC progression, proteomic analysis was performed. Total proteins were isolated through RIPA buffer and enzymatically cleaved using sequence-grade trypsin. The peptide mixtures underwent separation through nanoflow liquid chromatography and then measured with Q Exactive HF-X Mass Spectrometer (Thermo, USA). Raw files were converted, filtered, and normalized. Differentially expressed proteins were determined using standards including |fold change| > 1.5 and *P.*adj < 0.05. We utilized the Database for Annotation Visualization and Integrated Discovery (https://davidbioinformatics.nih.gov/) to perform functional enrichment.

### IF analysis

IF analysis was carried out to detect the SIA-IgG level. Cells received 15 min of fixation with 4% paraformaldehyde, before 10 min of 0.1% Triton X-100 permeabilization and additional 1 h of 5% bovine serum albumin blocking. After overnight primary antibody incubation against SIA-IgG at 4 °C, cells underwent another 1 h of secondary fluorescently labeled antibody incubation. Cell nuclei received 20 min of Hoechst staining (20 μg/ml, Invitrogen, USA). Finally, NIS-Elements Viewer (Nikon, Japan) was employed to scan the confocal images.

Multiplexed IF staining (Absin, China) was carried out to investigate the subcellular localization of SIA-IgG, VCP, and NDUFB6 via tyramide signal amplification (Absin, China). Cell fixation and permeabilization were performed following an identical IF protocol. The samples were then exposed to 15 min of 3% H_2_O_2_ treatment for blocking the endogenous peroxidase. Blocking, primary antibody incubation, and species-matched HRP-linked secondary antibody incubation were conducted as described in the IF analysis section. For the primary labeling step, the cell monolayer was completely covered by applying 100 μl of TYR fluorophore solution, prior to 5 min of incubation at ambient temperature. Secondary and tertiary labeling steps were conducted using alternative TYR fluorophores with target-specific antibodies after primary antibody stripping with 200 μl of IHC-staining-grade elution buffer for 10 min. Finally, cell nucleus staining and image scanning followed the detailed procedure described in the IF analysis section.

### Surface marker detection, ALDH analysis, and tumor sphere formation

For surface marker detection, the harvested cells subjected to centrifugation and resuspension in PBS at 1 × 10^6^ cells/100 μl. Cells were incubated with 20 μl of fluorophore-labeled primary antibodies at 4 °C for a 30-min duration or sequentially stained with unconjugated primary antibodies followed by species-matched secondary antibodies at 4 °C for a 0.5-h duration. Cells were later washed with PBS twice prior to resuspension in PBS (200 μl) and subjected to flow cytometric analysis via FACS Calibur. SIA-IgG^+^ cell sorting was completed with a BD FACS Aria SORP flow cytometer following the abovementioned antibody incubation.

The ALDEFLUOR assay kit (STEMCELL Technologies, USA) was employed to detect ALDH activity and sort CD44^+^/ALDH1^+^ cells. We incubated 1 × 10^6^ cells using activated ALDEFLUOR buffer supplemented with 5 μl of ALDEFLUOR DEAB buffer at 37 °C for 45 min. The samples were then employed to detect ALDH activity using FACS Calibur. For CD44^+^/ALDH1^+^ cell sorting, the samples were subsequently probed using PE-conjugated anti-CD44 antibody (20 μl) for 30 min at 4 °C. Cell pellets underwent resuspension with 0.5 ml of ALDEFLUOR buffer and maintained on ice for sorting negative and positive cells using BD FACS Aria SORP.

For tumor sphere formation analysis, OSCC cells (2 × 10^4^ cells/well) were suspended in tumor sphere medium in low-attachment 6-well plates (NEST Biotechnology, China), with medium supplementation at 5-d intervals with DMEM/F-12, 20 ng/ml epidermal growth factor, and 20 ng/ml basic fibroblast growth factor (Sino Biological Inc., China); 1× B-27 (Thermo, USA); and 1× N-2 (Thermo, USA). Spheres (>70 μm) were counted microscopically after 14-d culture.

### Transmission electronic microscopy

After fixation in 2.5% glutaraldehyde, cells underwent dehydration using gradient ethanol dehydration (30%, 50%, 70%, 80%, 90%, 100%, and 100%). A mixture of acetone and epoxy resin was employed to infiltrate samples. The infiltrated samples then received embedding within Embed-812 epoxy resin and 48 h of polymerization at 60 °C; 70-nm ultrathin sections were obtained with an ultramicrotome and loaded onto copper grids before double staining using lead citrate and uranyl acetate. Lastly, a transmission electron microscope (JEM-100CX, Japan) was used for sample examination at 100 kV.

### Mitochondrial stress assay

Seahorse XF Mito Stress Test Kits (Agilent, USA) were employed to analyze mitochondrial stress following a specific protocol. In brief, 1.5 × 10^4^ cells/well were plated into XFe96 microplates before overnight incubation at 37 °C. Probe plates subjected to hydration using XF Calibrant. Wells were washed twice with assay medium (Seahorse XF DMEM pH 7.4, 10 mM glucose, 1 mM pyruvate, and 2 mM glutamine), and the excess washing solution was discarded to maintain a final volume of 180 μl. Then, oligomycin (1.5 μM), carbonyl cyanide 4-(trifluoromethoxy) phenylhydrazone (FCCP, 1 μM), and rotenone/antimycin A (0.5 μM) were loaded, respectively. Finally, metabolic parameters were recorded. OCRs were determined by the Agilent Seahorse Wave Pro software.

### In vivo experiment

All animal experiments gained approval from the Institutional Animal Care and Use Committee of Peking University Health Science Center, according to the Animal Ethical and Welfare Committee protocol (approval number: LA2023169). In the common xenograft tumor model, we used healthy 4-week-old male BALB/c nude mice and added 100 μl of PBS to resuspend 5 × 10^6^ cells. For the extreme dilution tumor model, we used healthy 4-week-old male BALB/c nude mice, too, and 100 μl of PBS was introduced to resuspend 500, 1,000, and 10,000 cells, which were later added to an equal volume of Matrigel (NEST Biotechnology, China). The prepared cell suspension was subcutaneously injected into the back of mice. The following formula was utilized to measure tumor size: *V* = length × width^2^/2. The ELDA software (http://bioinf.wehi.edu.au/software/elda/) was used to calculate the stem cell frequency and tumor formation rates. To observe drug-induced tumor growth inhibition, we constructed xenograft tumor and syngeneic tumor models using BALB/c nude mice (4 weeks old, male) and C57BL/6 mice (4 weeks old, male), respectively. The corresponding drugs were injected through the tail vein at 3-d intervals after the tumor volume reached about 50 mm^3^. All mice were anesthetized after the experiment, while samples were obtained to perform subsequent experiments.

### Blood routine and blood biochemistry test

Blood routine and blood biochemistry tests were performed to detect the impact of drugs on various bodily functions; 100 to 200 μl of blood was taken from the eyeballs of nude mice and added into a 1-ml anticoagulant tube. Whole blood was used to conduct blood routine tests, which included indicators such as white blood cell, red blood cell, platelet, and lymphocyte (Lymph%) counts. Blood underwent 15 min of centrifugation to collect supernatants to obtain serum for biochemical testing, including albumin, alanine aminotransferase, aspartate aminotransferase, total bilirubin, blood urea nitrogen, total bile acid, uric acid, and creatinine.

### HE staining and IHC staining

Tissue samples were processed through 24 h of 4% paraformaldehyde fixation at 4 °C, paraffin embedding, and slicing into 4-μm-thick slides, followed by deparaffinization in xylene and rehydration using descending ethanol concentrations. The slides were subjected to HE staining. In IHC, slides underwent heat-induced antigen retrieval and endogenous peroxidase blocking before overnight incubation at 4 °C using primary antibody. Subsequently, slides were probed with HRP-conjugated secondary antibodies and visualized via diaminobenzidine. Finally, an Olympus BX51 microscope (Olympus, Japan) was used for imaging. The number of positive cells and the staining intensity were calculated by randomly selecting 5 regions. The percentage classification of stained cells in each area is as follows: 0, negative; 1, 1% to 25%; 2, 26% to 50%; and 3, 51% to 100%. The staining intensity classification is as follows: 0, no signal; 1, light brown indicates a low-intensity signal; 2, brown indicates a medium-intensity signal; and 3, dark brown indicates a high-intensity signal. The score of each area is calculated as the percentage grade of stained cells × the grade of staining intensity. If the final score is 0 to 1, RP215-SIA-IgG is negative (−); if the score is 2 to 3, RP215-SIA-IgG is lowly expressed (+); and if the scores are 4 to 6 and 7 to 9, RP215-SIA-IgG is highly expressed (++ and +++).

### Statistical analyses

Data visualization was completed with the GraphPad Prism 7.0 and R software (v4.4.3). Statistical analyses were conducted via SPSS 25.0. Between-group normally distributed data were examined through Student *t* tests, whereas multigroup analyses employed one-way analysis of variance. Nonnormally distributed data were analyzed with the Wilcoxon rank-sum test. *P* < 0.05 stood for significant differences.

## Data Availability

All data supporting the present work can be obtained from the corresponding authors according to requirements.
