# Supplementary material for: Sialylated Immunoglobulin G Promotes the Malignant Progression of Oral Squamous Cell Carcinoma through VCP-Mediated NDUFB6 Stabilization Regulated Mitochondrial Oxidative Phosphorylation
Source: Research (Wash D C). 2025 Dec 12;8:0985. doi: 10.34133/research.0985 (PMC13248703; doi:10.34133/research.0985)

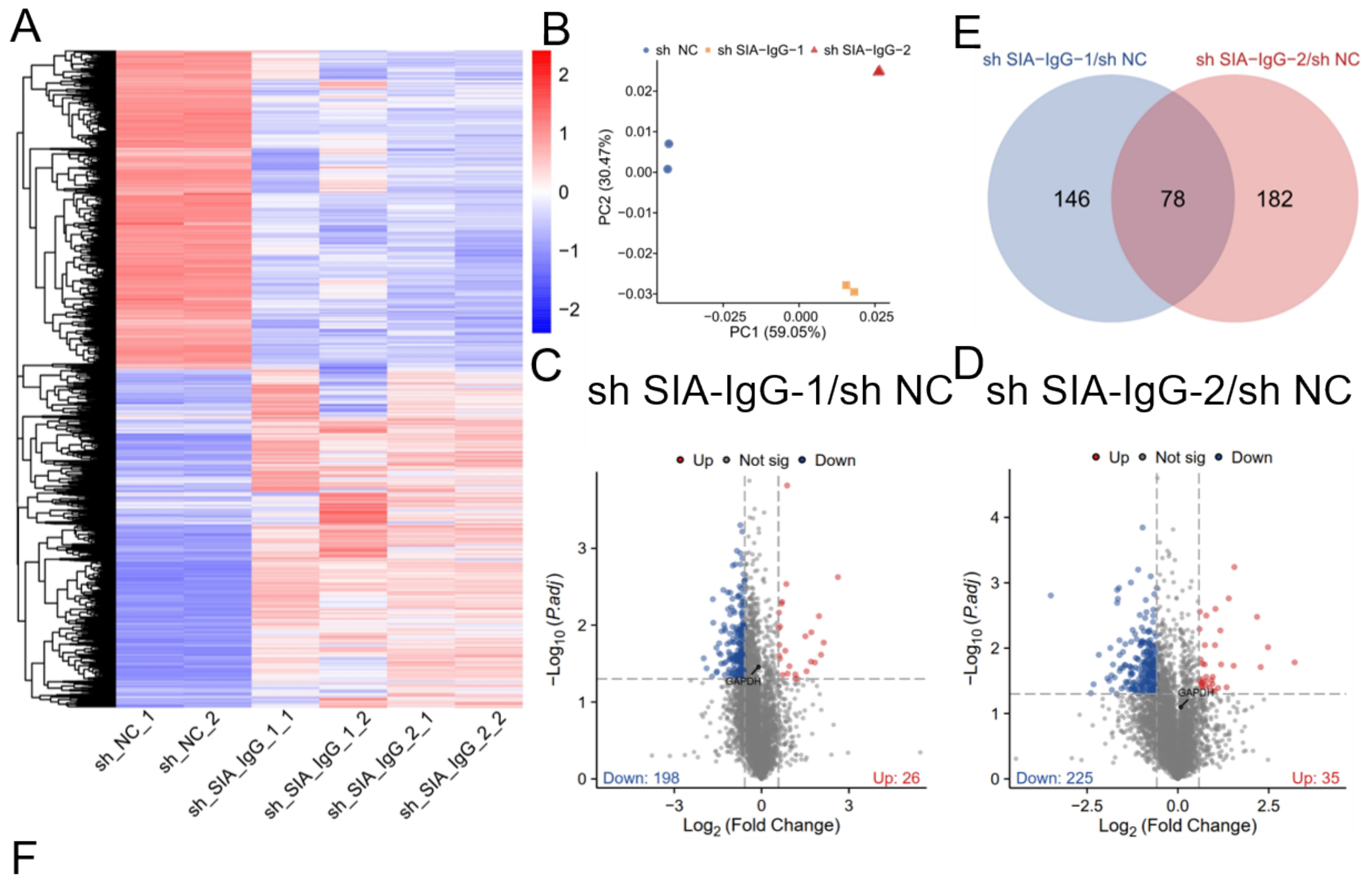

F

ID	Term	Count	P Value
hsa04068	FoxO signaling pathway	3	0.011721
hsa00190	Oxidative phosphorylation	3	0.012165
hsa03040	Spliceosome	5	0.015873
hsa03010	Ribosome	3	0.017778
hsa05014	Amyotrophic lateral sclerosis	6	0.022682
hsa05322	Systemic lupus erythematosus	4	0.023365
hsa05131	Shigellosis	5	0.02429
hsa01100	Metabolic pathways	9	0.039427
hsa05034	Alcoholism	4	0.051976
hsa05208	Chemical carcinogenesis - reactive oxygen species	4	0.078201

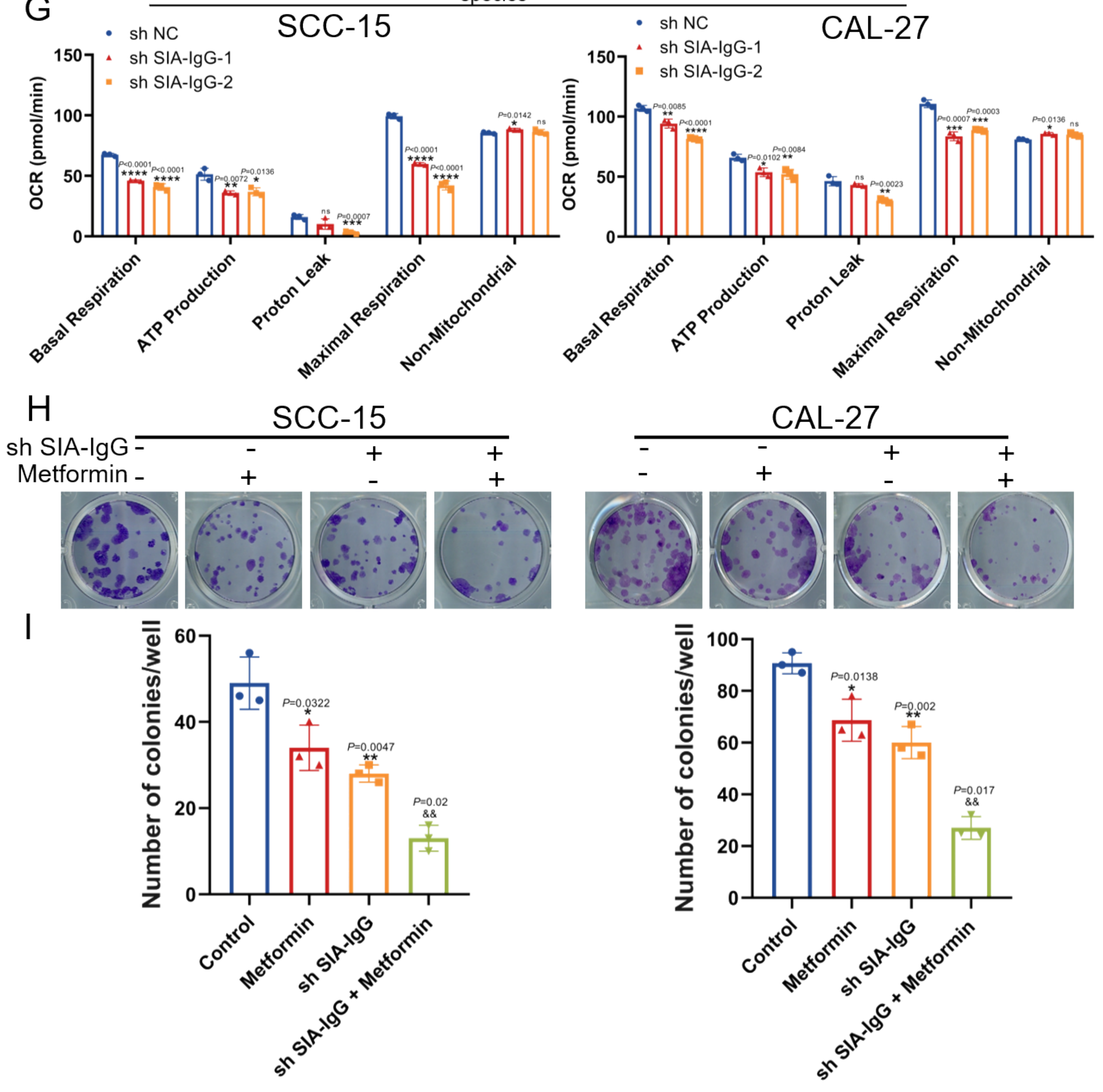

Supplement: Supplementary 1 — Figs. S1 to S11 [file research.0985.f1.zip › Figure S5.pdf]
