# Supplementary material for: Sialylated Immunoglobulin G Promotes the Malignant Progression of Oral Squamous Cell Carcinoma through VCP-Mediated NDUFB6 Stabilization Regulated Mitochondrial Oxidative Phosphorylation
Source: Research (Wash D C). 2025 Dec 12;8:0985. doi: 10.34133/research.0985 (PMC13248703; doi:10.34133/research.0985)
Supplement: Supplementary 1 — Figs. S1 to S11 [file research.0985.f1.zip › Figure S6.pdf]

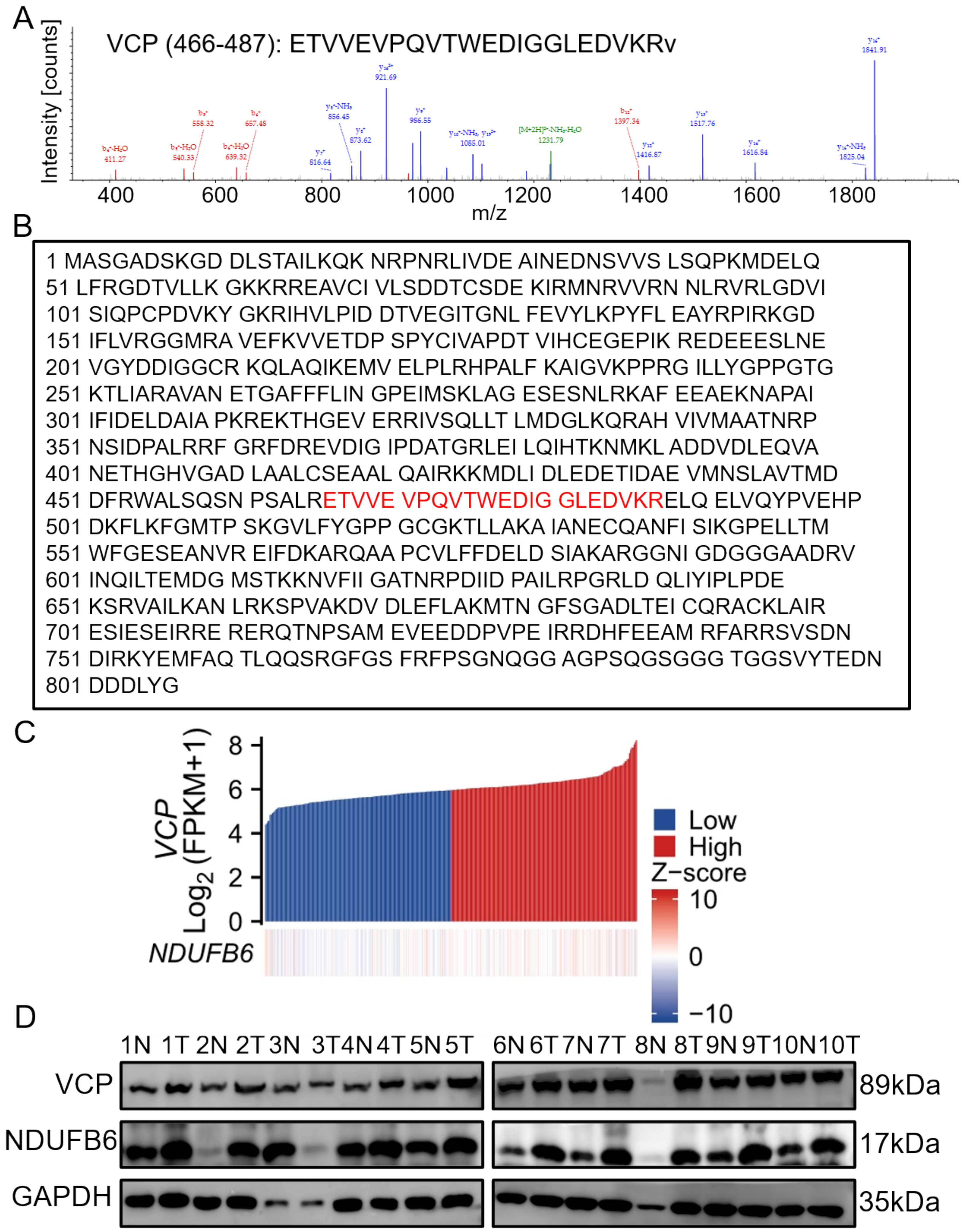
A
VCP (466-487): ETVVEVPQVTWEDIGGLEDVKRv
Intensity [counts]
400
600
800
1000
1200
1400
1600
1800
m/z
B
1 MASGADSKGD DLSTAILKQK NRPNRLIVDE AINEDNSVVS LSQPKMDELQ
51 LFRGDTVLLK GKKRREAVCI VLSDDTCSDE KIRMNRVVRN NLRVRLGDVI
101 SIQPCPDVKY GKRIHVLPID DTVEGITGNL FEVYLKPYFL EAYRPIRKGD
151 IFLVRGGMRA VEFKVVETDP SPYCIVAPDT VIHCEGEPIK REDEEESLNE
201 VGYDDIGGCR KQLAQIKEMV ELPLRHPALF KAIGVKPPRG ILLYGPPGTG
251 KTLIARAVAN ETGAFFFLIN GPEIMSKLAG ESESNLRKAF EEAEKNAPAI
301 IFIDELDAIA PKREKTHGEV ERRIVSQLLT LMDGLKQRAH VIVMAATNRP
351 NSIDPALRRF GRFDREVDIG IPDATGRLEI LQIHTKNMKL ADDVDLEQVA
401 NETHGHVGAD LAALCSEAAL QAIRKKMDLI DLEDETIDAE VMNSLAVTMD
451 DFRWALSQSN PSALRETVVE VPQVTWEDIG GLEDVKRELQ ELVQYPVEHP
501 DKFLKFGMTP SKGVLFYGPP GCGKTLLAKA IANECQANFI SIKGPELLTM
551 WFGESEANVR EIFDKARQAA PCVLFFDELD SIAKARGGNI GDGGGAADRV
601 INQILTEMDG MSTKKNVFII GATNRPDIID PAILRPGRLD QLIYIPLPDE
651 KSRVAILKAN LRKSPVAKDV DLEFLAKMTN GFSGADLTEI CQRACKLAIR
701 ESIESEIRRE RERQTNPSAM EVEEDDPVPE IRRDHFEEAM RFARRSVSDN
751 DIRKYEMFAQ TLQQSRGFGS FRFPSGNQGG AGPSQGSGGG TGGSVYTEDN
801 DDDLYG
C
VCP
Log2 (FPKM+1)
0
2
4
6
8
Low
High
Z−score
10
0
−10
NDUFB6
D
1N 1T 2N 2T 3N 3T 4N 4T 5N 5T
6N 6T 7N 7T 8N 8T 9N 9T 10N 10T
VCP
NDUFB6
GAPDH
89kDa
17kDa
35kDa
