# Supplementary material for: Sialylated Immunoglobulin G Promotes the Malignant Progression of Oral Squamous Cell Carcinoma through VCP-Mediated NDUFB6 Stabilization Regulated Mitochondrial Oxidative Phosphorylation
Source: Research (Wash D C). 2025 Dec 12;8:0985. doi: 10.34133/research.0985 (PMC13248703; doi:10.34133/research.0985)
Supplement: Supplementary 1 — Figs. S1 to S11 [file research.0985.f1.zip › Revised Supplementary Figure (with Highlighted Changes).docx]

Supplementary Figure S1.


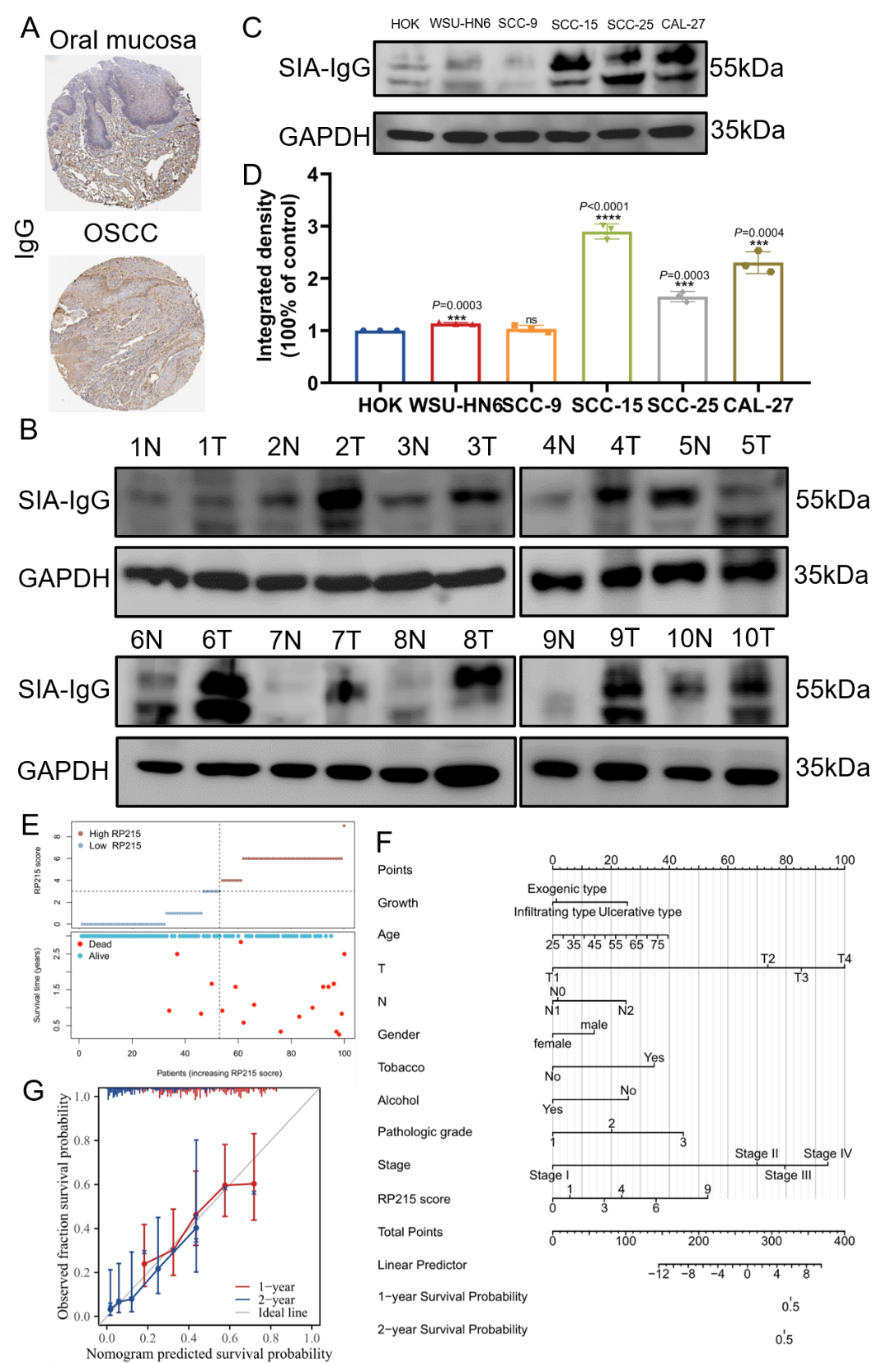


Figure S1. Upregulation of SIA-IgG is positively correlated with poor prognosis. (A) The IHC representative images of IgG in the HPA database. (B) The expression of SIA-IgG in OSCC tissues (T) and adjacent normal tissues (N); *n* = 10 per group. (C) The expression level of SIA-IgG in different cell lines was detected by western blot. (D) Quantitative analysis of (C); *n* = 3 per group. (E) Distribution map of RP215 staining scores (up) and survival status (down); *n*= 53 in the low RP215 group, *n* = 47 in the high RP215 group. (F) Prognostic nomogram. (G) Internal correction curve of the prognostic nomogram. * indicates differences between OSCC cells and HOK groups, ns, *P* > 0.05; ***, *P* < 0.001; ****, *P* < 0.0001 by one-way analysis of variance with post hoc test.

Supplementary Figure S2.


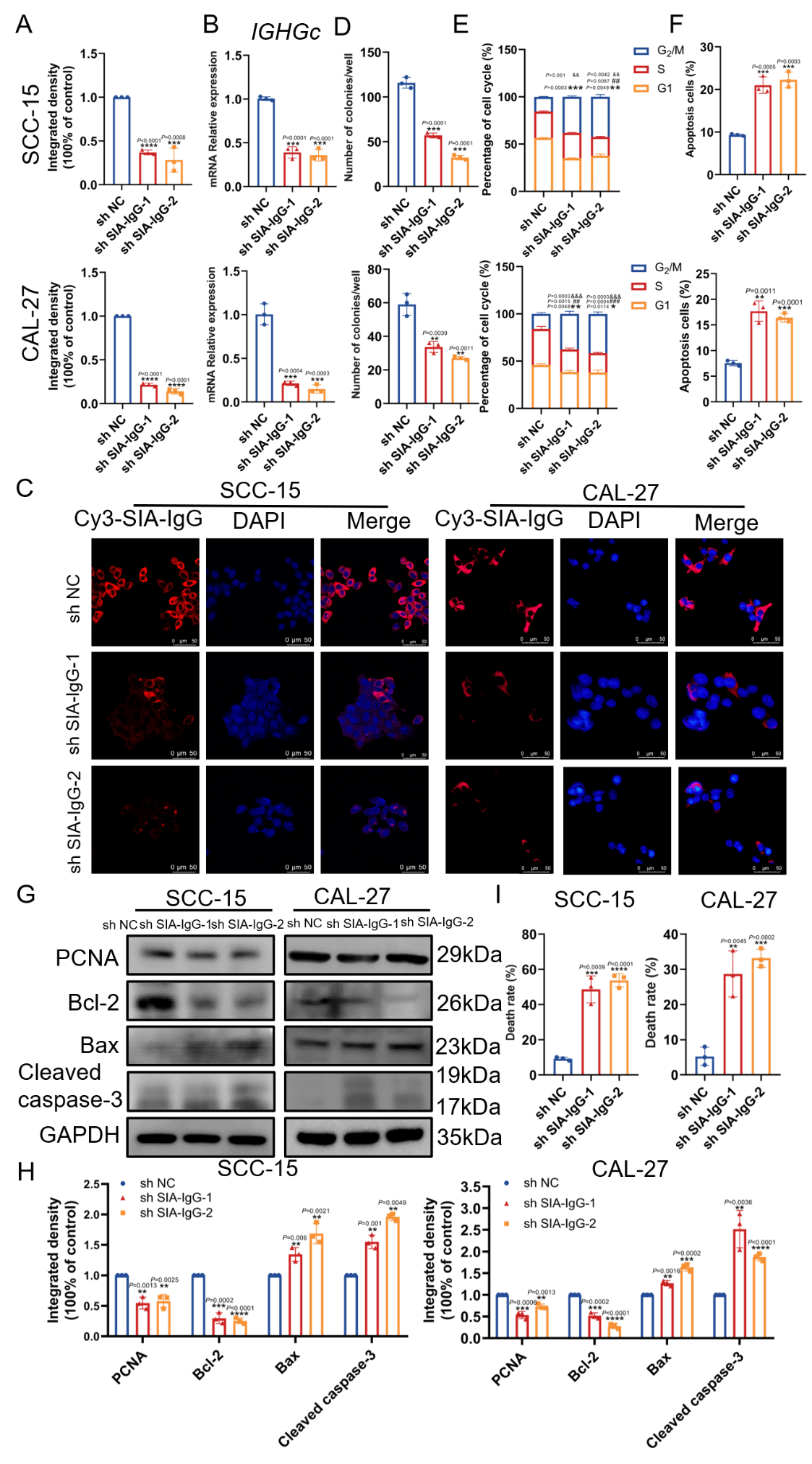


Figure S2. SIA-IgG promotes malignant progression of OSCC. (A-C) SCC-15 and CAL-27 cells were transfected with two anti-SIA-IgG shRNA, and the knockdown effects were detected by western blott (*n* = 3 per group), RT-PCR analysis (*n* = 3 per group), and IF staining (magnification ×40; scale bar, 50 µm). (D) Statistical graphs of cell clone formation in each group; *n* = 3 per group. (E) Cell cycle statistical graphs of each group; *n* = 3 per group. (F) Statistical graphs of apoptosis in each group; *n* = 3 per group. (G) Western blot analysis of PCNA, Bcl-2, Bax, and cleaved caspase-3 expression levels in SCC-15 cells (left) and CAL-27 cells (right). (H) Quantitative analysis of (G); *n* = 3 per group. * indicates the differences between G1 phase or other biological behaviors in knockdown groups and control group, # indicates the differences between S phase in knockdown groups and control group, & indicates the differences between the G_2_/M phase in knockdown groups and control group. *, *P* < 0.05; ** ## &&, *P* < 0.01; *** ### &&&, *P* < 0.001; ****, *P* < 0.0001 by one-way analysis of variance with post hoc test.

Supplementary Figure S3.


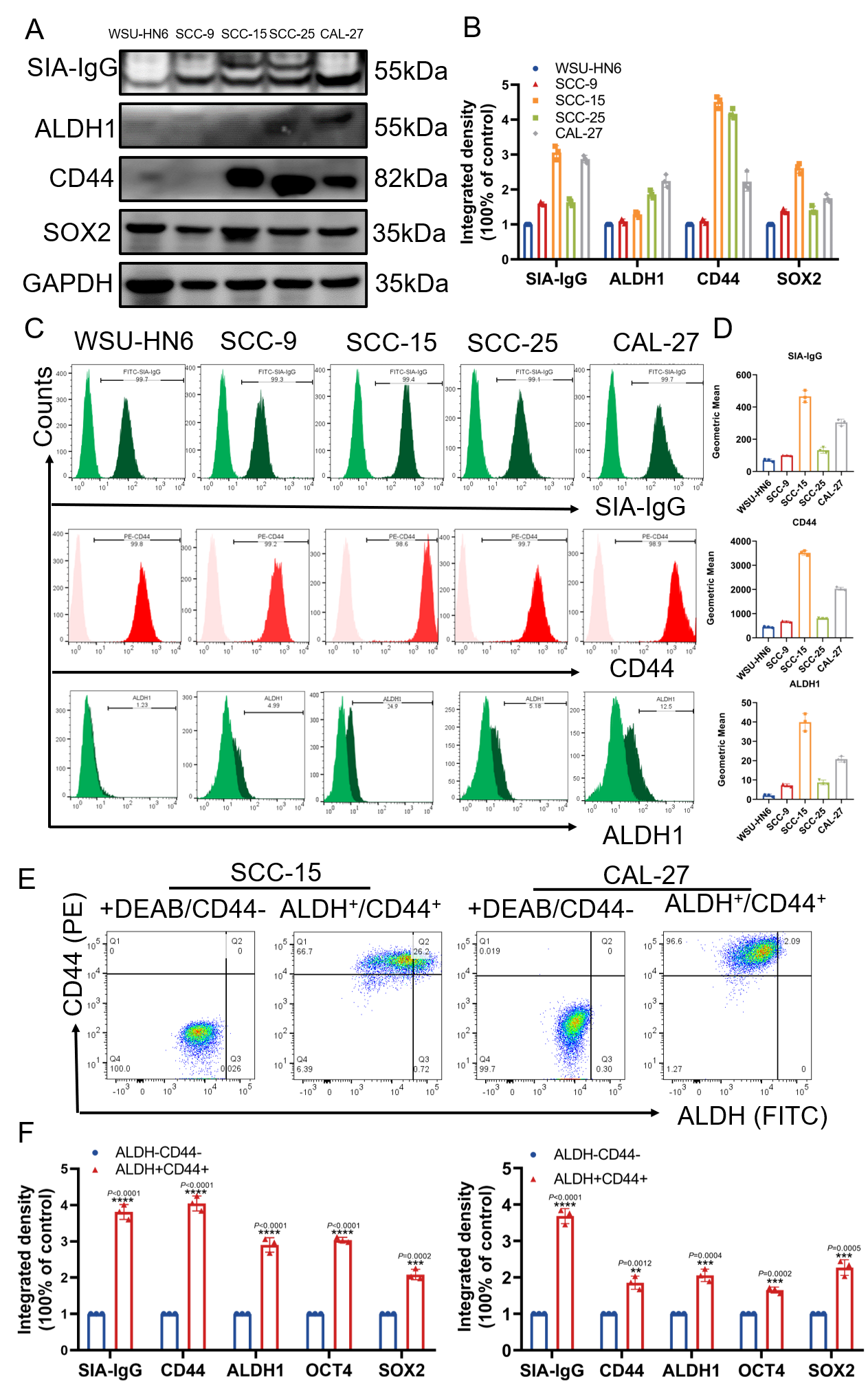


Figure S3. SIA-IgG has a positive correlation with the tumor stemness in OSCC. (A) The protein expression levels of SIA-IgG and CSC markers in OSCC cells. (B) Quantitative analysis of (A); *n* = 3 per group. (C-D) Flow cytometry analyses of SIA-IgG and CSC markers in OSCC cells (B) and statistical analysis of fluorescence intensity (C); *n* = 3 per group. (E) Flow cytometry was used to sort CD44^+^/ALDH^+^ and negative cells in SCC-15 and CAL-27 cells. (F) Quantitative analysis of (Fig. 3A); *n* = 3 per group. **, *P* < 0.01; ***, *P* < 0.001; ****, *P* < 0.0001 by unpaired *t* test.

Supplementary Figure S4.


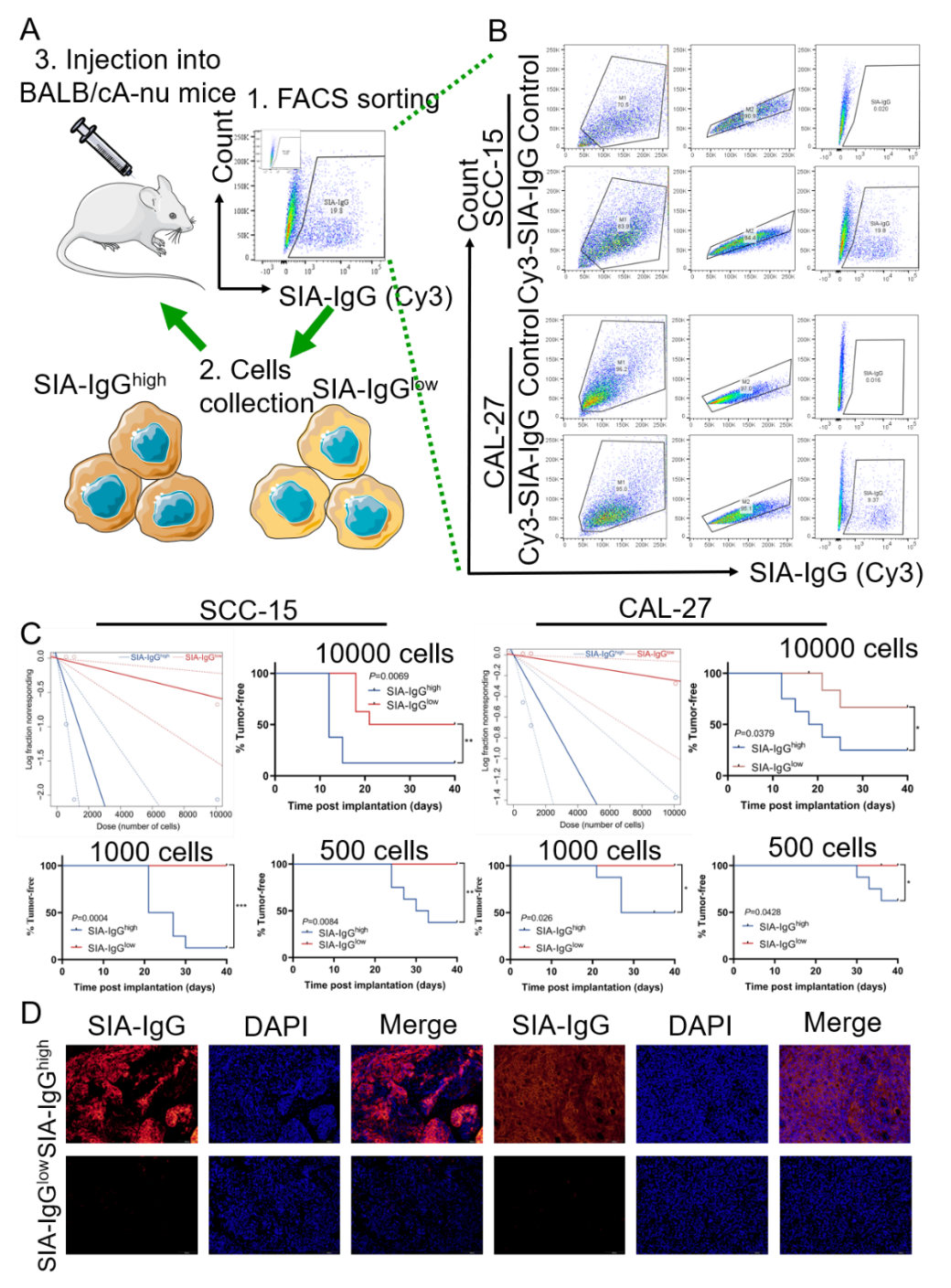


Figure S4. SIA-IgG promotes the tumorigenesis ability of a small number of OSCC cells in vivo. (A) The overall flowchart of the experiment. (B) After Cy3-labeling SIA-IgG, SIA-IgG^high^ cells and SIA-IgG^low^ cells in SCC-15 and CAL-27 were sorted by flow cytometry. (C) The tumor formation curves of each group during the observation time; *n* = 8 per group. (D) The expression intensity of SIA-IgG in the SIA-IgG^high/low^ group was detected by IF. SIA-IgG was labeled with Cy3 (red), and the cell nucleus was labeled with DAPI (blue) (magnification ×20; scale bar, 100 µm). *, *P* < 0.05; **, *P* < 0.01; ***, *P* < 0.001 by log-rank test.

Supplementary Figure S5.


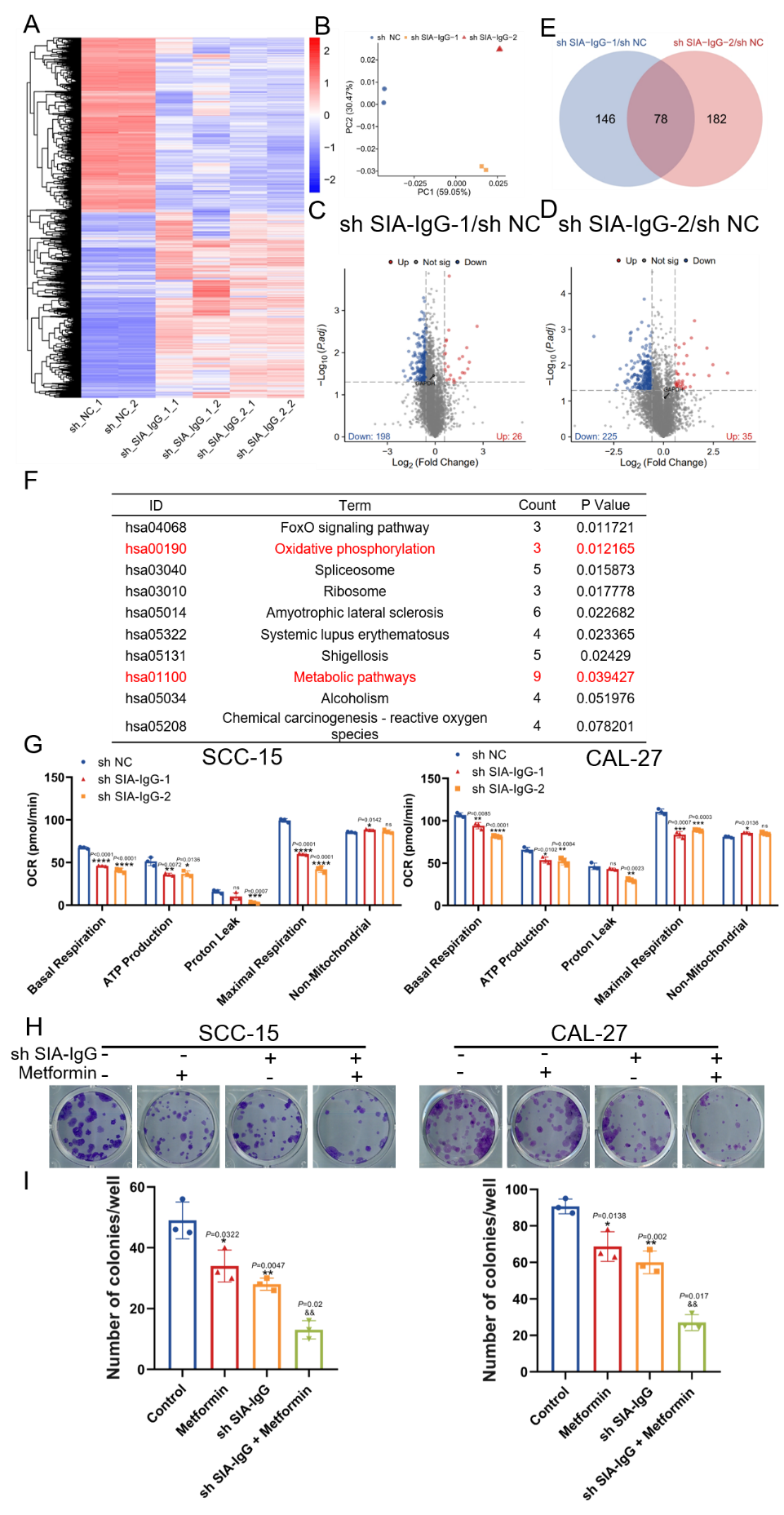


Figure S5. SIA-IgG can activate the OXPHOS pathway in OSCC. (A) Proteomic profiling of protein expression heat map of SCC-15 cells in each group; *n* = 2 per group. (B) PCA analysis diagrams of each group; *n* = 2 per group. (C-D) The volcano plot of differential proteins. The blue and red dots represent proteins with downregulated or expression in knockdown group, respectively. The screening criteria are |log_2_ (fold change) | > 0.58 and *P*.adj < 0.05. (E) The overlapping Venn diagram of the differential proteins in the two knockdown groups and control group. (F) KEGG enriches specific content. (G) Statistical chart of OCR various parameters in SCC-15 cells (left) and CAL-27 cells (right); *n* = 3 per group. (H) Clone images for both cell lines. (I) Statistical graphs of cell clone formation in each group; *n* = 3 per group. * indicates the comparison between the control group and the SIA-IgG knockdown group or metformin group, & indicates the comparison between the SIA-IgG knockdown group and the SIA-IgG knockdown group + metformin group. ns, *P* > 0.05; *, *P* < 0.05; ** &&, *P* < 0.01; ***, *P* < 0.001; ****, *P* < 0.0001 by one-way analysis of variance with post hoc test.

Supplementary Figure S6.


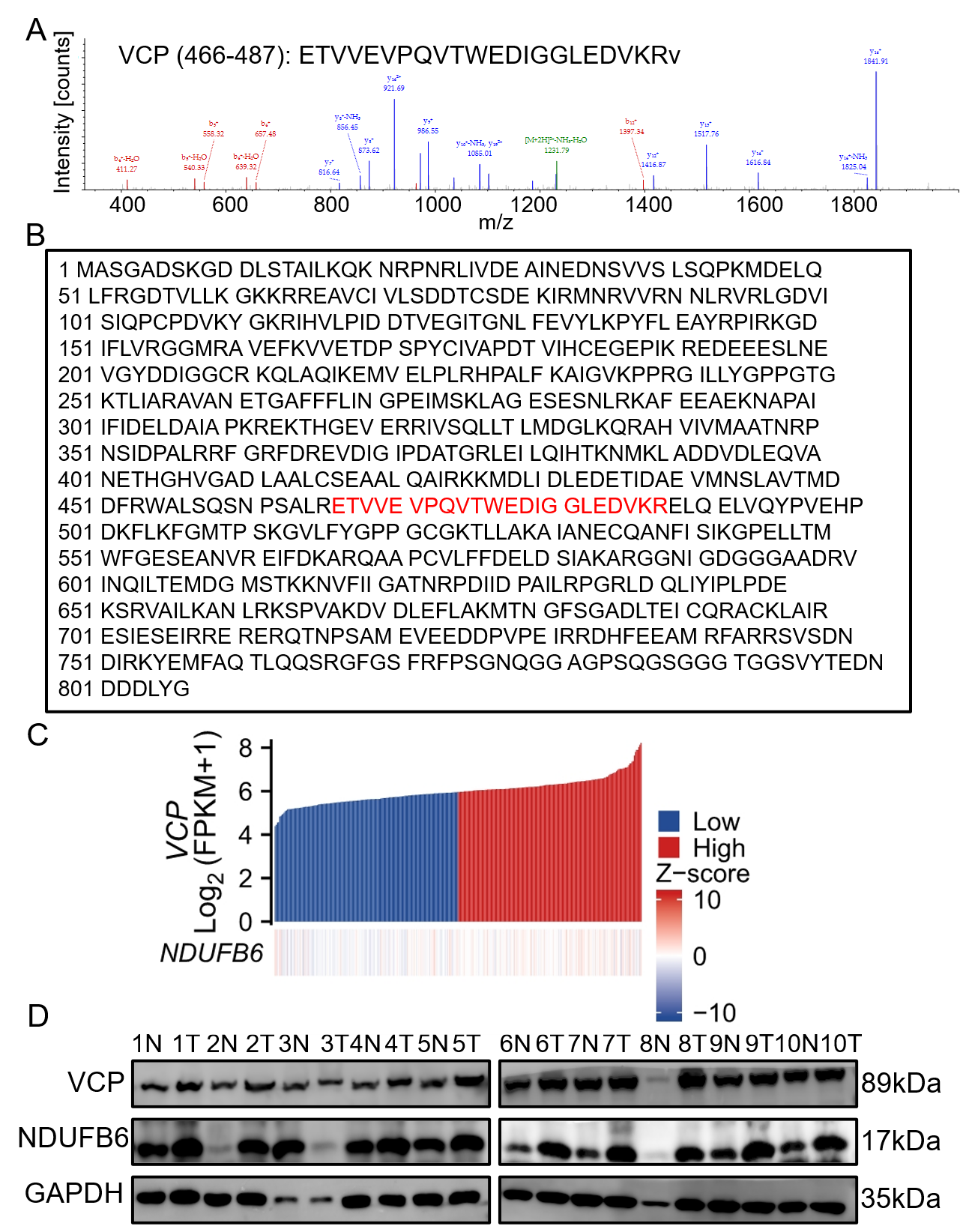


Figure S6. SIA-IgG, VCP and NDUFB6 interact in OSCC cells. (A) By analyzing the mass differences in the y and b fragment ion series, the representative MS/MS spectrum of VCP was used to identify its amino acid sequence as ETVVEVPQVTWEDIGGLEDVKR. (B) In the protein sequence of VCP, the matched peptides are highlighted in red bold type. (C) Among OSCC patients in the TCGA database, the expression heat maps of VCP and NDUFB6 (*n* = 330). (D) The expression of VCP and NDUFB6 in OSCC tissues (T) and adjacent normal tissues (N); *n* = 10 per group.

Supplementary Figure S7.


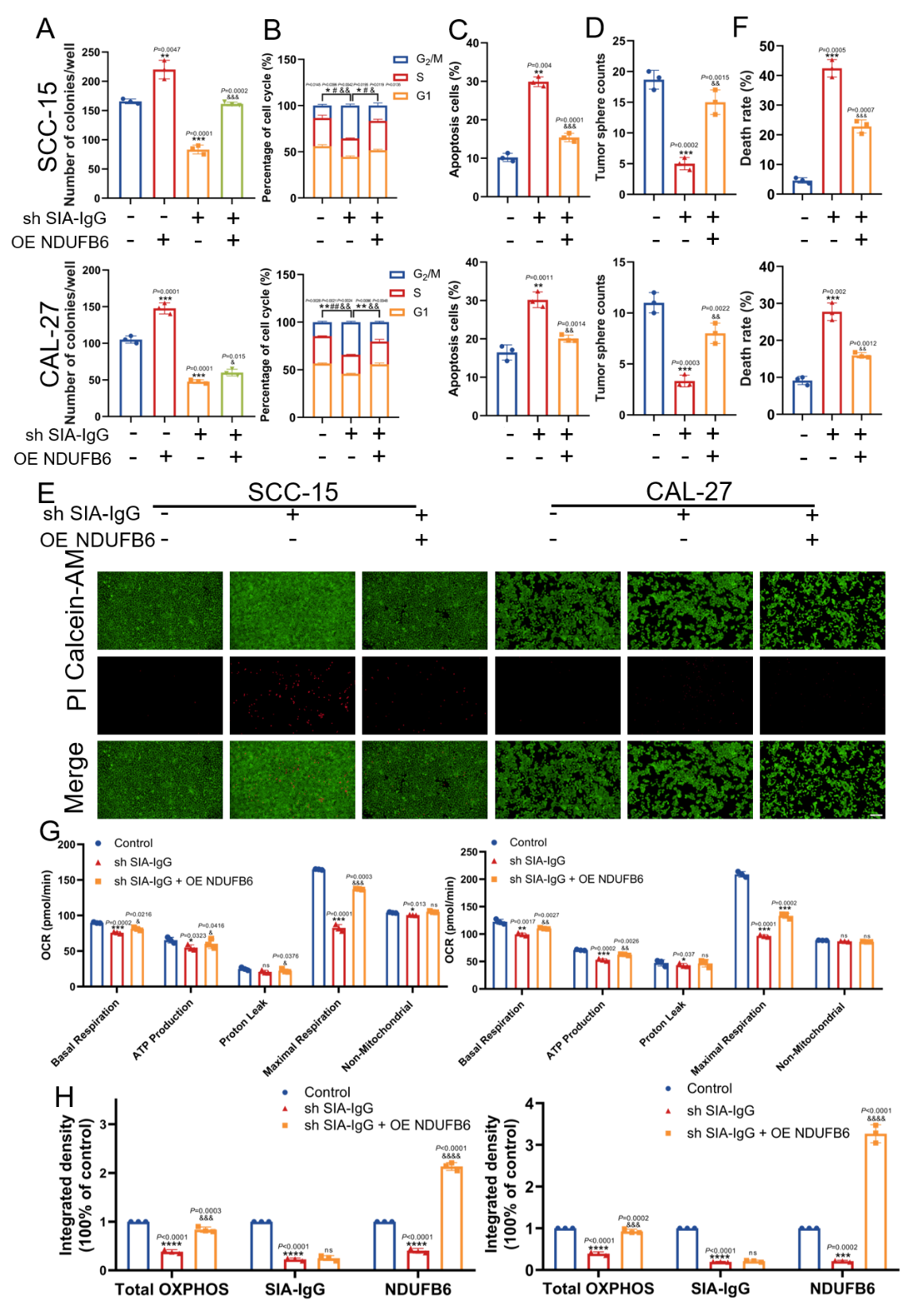


Figure S7. SIA-IgG can enhance the expression of NDUFB6 and thereby activate OXPHOS in OSCC. (A-G) Statistical of clone formation, statistical of cycle distribution, statistical of apoptosis, statistical of tumor spheres, calcein-AM/PI staining plots (magnification ×10; scale bar, 200 µm), statistical of death rate, and statistical chart of OCR various parameters in each group, respectively; *n* = 3 per group. (H) Quantitative analysis of (Fig. 8G); *n* = 3 per group. In figures A, C, D, F, G, and H * indicates the comparison between the control group and the SIA-IgG knockdown group or NDUFB6 overexpression group, & indicates the comparison between the SIA-IgG knockdown group and the SIA-IgG knockdown group + NDUFB6 overexpression group. In figure B, * indicates the differences between G1 phase in the two groups, # indicates the differences between S phase in the two groups, & indicates the differences between the G_2_/M phase in the two groups. ns, *P* > 0.05; * # &, *P* < 0.05; ** ## &&, *P* < 0.01; *** &&&, *P* < 0.001; **** &&&&, *P* < 0.0001 by one-way analysis of variance with post hoc test.

Supplementary Figure S8.


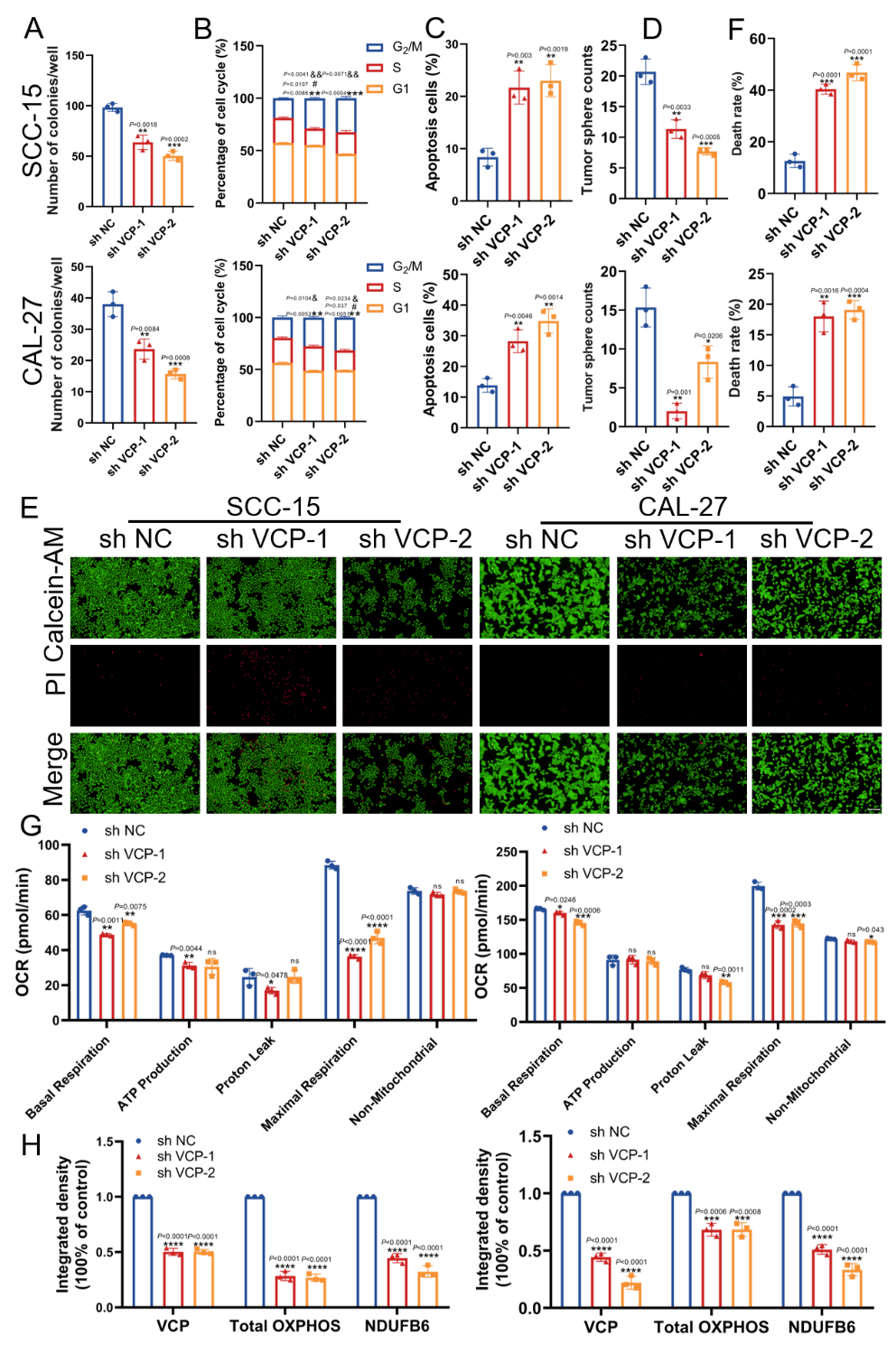


Figure S8. VCP promotes malignant progression of OSCC. (A-G) Statistical of clone formation, statistical of cycle distribution, statistical of apoptosis, statistical of tumor spheres, calcein-AM/PI staining plots (magnification ×10; scale bar, 200 µm), statistical of death rate, and statistical chart of OCR various parameters in each group, respectively; *n* = 3 per group. (H) Quantitative analysis of (Fig. 9H); *n* = 3 per group. In figure B, * indicates the differences between G1 phase in knockdown groups and control group, # indicates the differences between S phase in knockdown groups and control group, & indicates the differences between the G_2_/M phase in knockdown groups and control group. In figures A, C, D, F, G, and H * indicates the comparison between the control group and the VCP knockdown groups. ns, *P* > 0.05; * # &, *P* < 0.05; ** &&, *P* < 0.01; ***, *P* < 0.001; ****, *P* < 0.0001 by one-way analysis of variance with post hoc test.

Supplementary Figure S9.


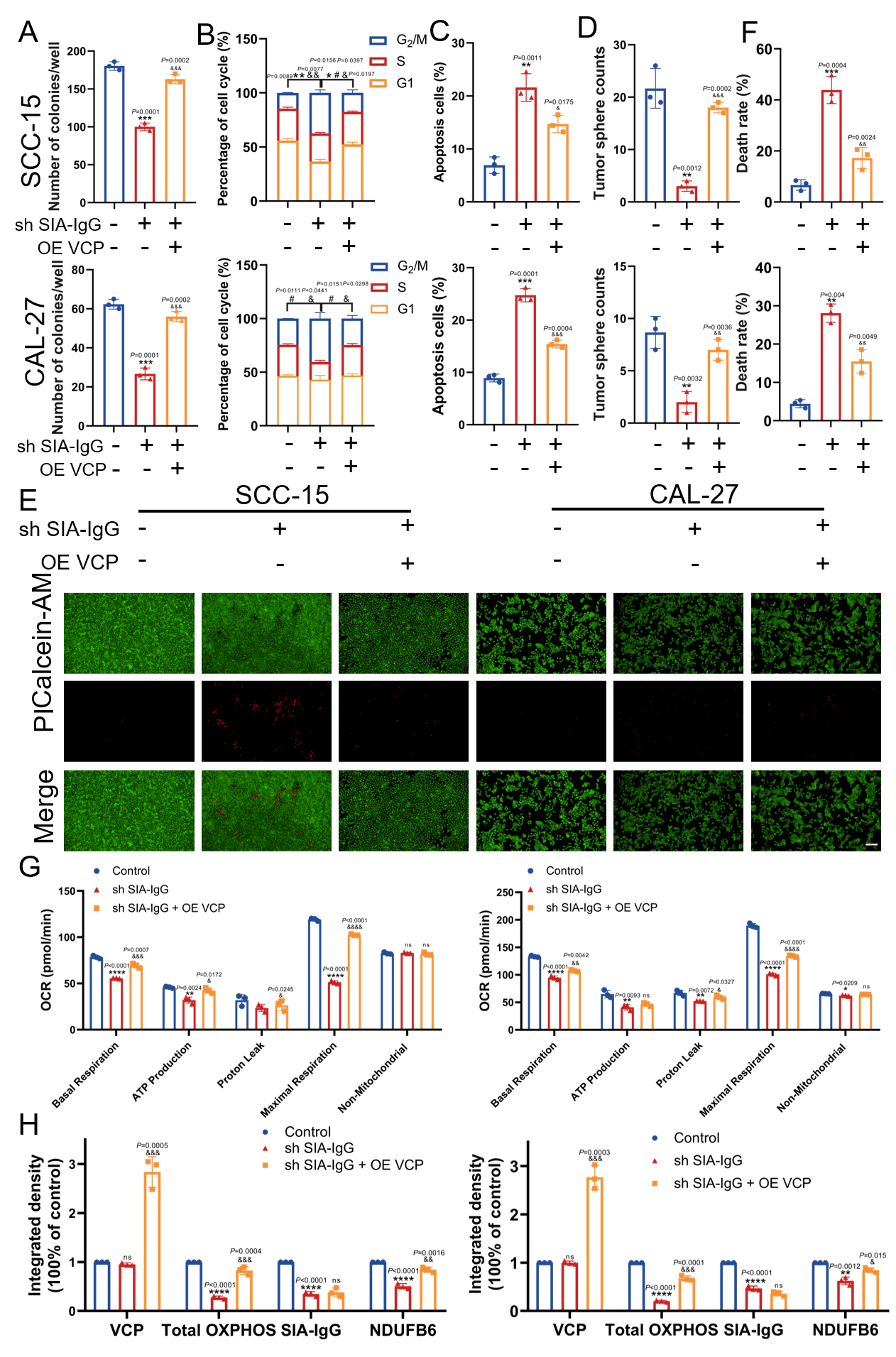


Figure S9. Overexpression of VCP suppresses the pro-malignant progression effect of SIA-IgG. (A-G) Statistical of clone formation, statistical of cycle distribution, statistical of apoptosis, statistical of tumor spheres, calcein-AM/PI staining plots (magnification ×10; scale bar, 200 µm), statistical of death rate, and statistical chart of OCR various parameters in each group, respectively; *n* = 3 per group. (H) Quantitative analysis of (Fig. 10G); *n* = 3 per group. In figures A, C, D, F, G, and H, * indicates the comparison between the control group and the SIA-IgG knockdown group, & indicates the comparison between the SIA-IgG knockdown group and the SIA-IgG knockdown group + VCP overexpression group. In figure B, * indicates the differences between G1 phase in the two groups, # indicates the differences between S phase in the two groups, & indicates the differences between the G_2_/M phase in the two groups. ns, *P* > 0.05; * # &, *P* < 0.05; ** &&, *P* < 0.01; *** &&&, *P* < 0.001; **** &&&&, *P* < 0.0001 by one-way analysis of variance with post hoc test.

Supplementary Figure S10.


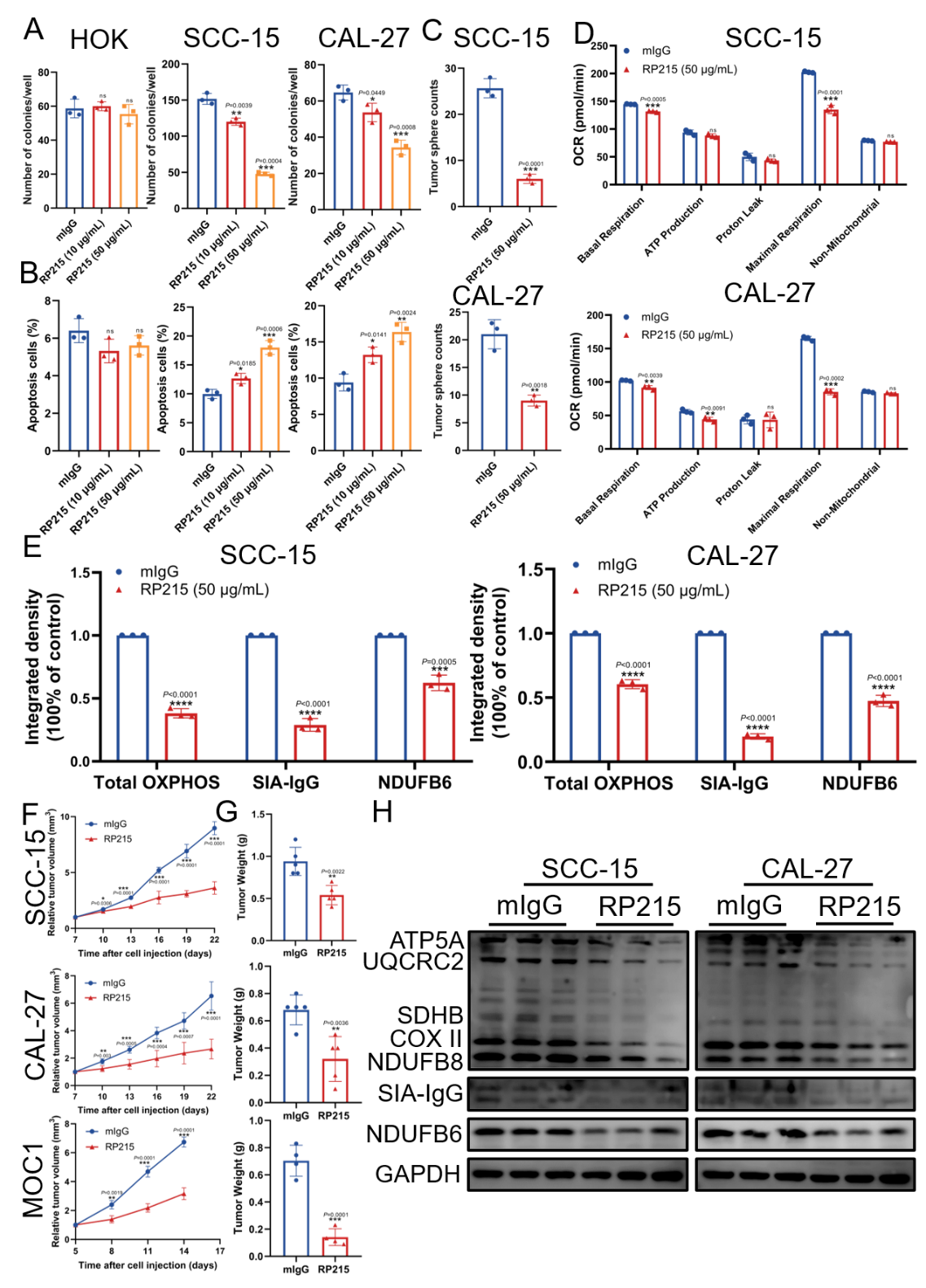


Figure S10. RP215 inhibits malignant progression of OSCC. (A-D) Statistical of clone formation, statistical of apoptosis, statistical of tumor spheres, and statistical chart of OCR various parameters in each group, respectively; *n* = 3 per group. (E) Quantitative analysis of (Fig. 11F); *n* = 3 per group. (F-G) Tumor volume and tumor weight of the mice injection of SCC-15 (up) (*n* = 5 per group), CAL-27 (middle) (*n* = 5 per group), and MOC1 (bottom) (*n* = 4 per group) cells, respectively. (H) Western blot analysis of tumor lysates for indicated proteins. * indicates the comparison between the RP215 group and the mIgG group. ns, *P* > 0.05; *, *P* < 0.05; **, *P* < 0.01; ***, *P* < 0.001; ****, *P* < 0.0001 by one-way analysis of variance or unpaired *t* test as appropriate.

Supplementary Figure S11.


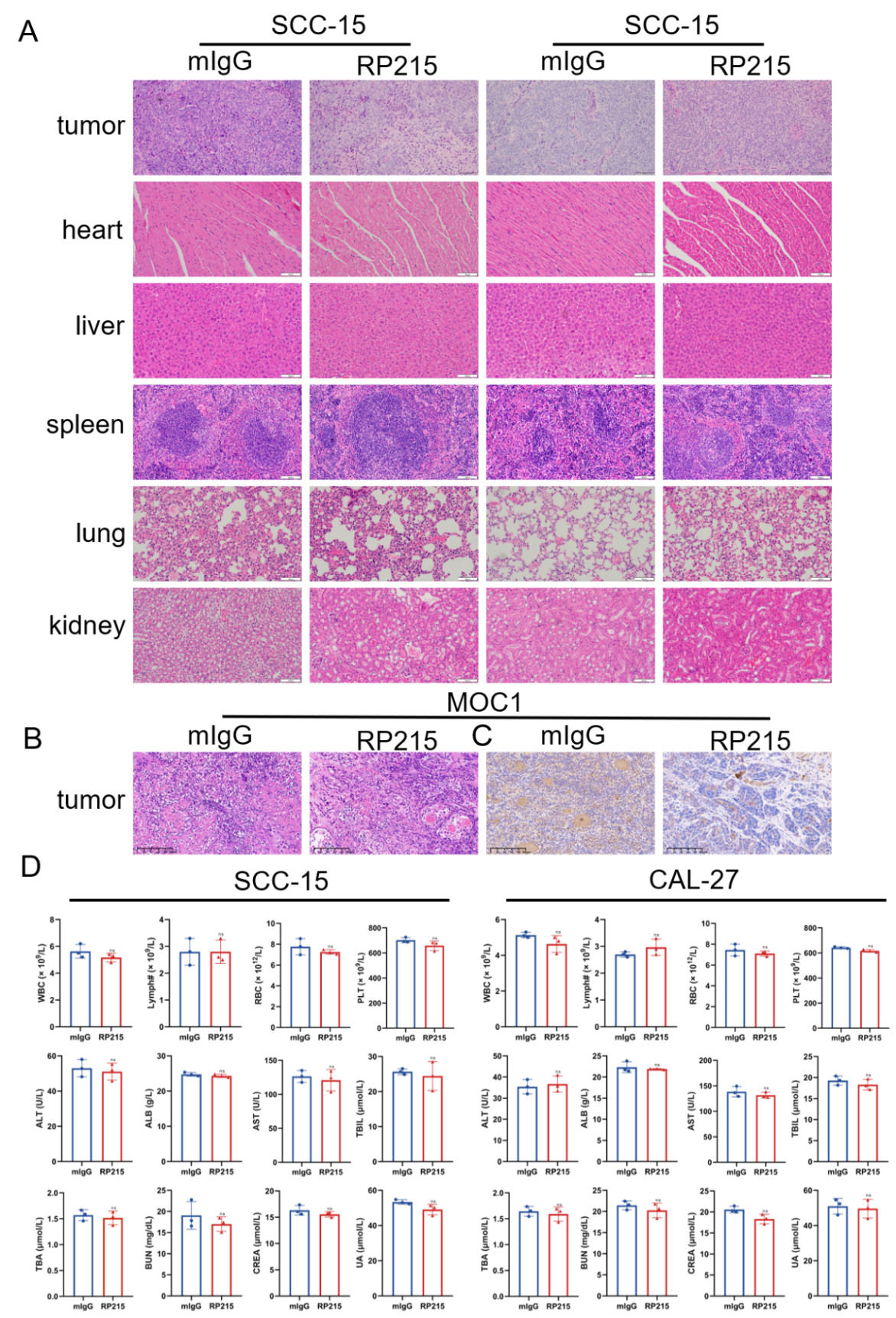


Figure S11. RP215 has no damage to the various functions of mice. (A) HE staining images of the tumor, hearts, livers, spleens, lungs and kidneys of mice in each group (magnification ×20; scale bar, 100 µm). (B) HE staining images of the tumors formed of MOC1 cells (magnification ×20; scale bar, 100 µm). (C) IHC staining images of the tumors formed of MOC1 cells (magnification ×20; scale bar, 100 µm). (D) Statistical charts of blood routine and various blood biochemical indicators in each group. ns, *P* > 0.05 by unpaired *t* test.
