# Supplementary figures and images for: Sialylated Immunoglobulin G Promotes the Malignant Progression of Oral Squamous Cell Carcinoma through VCP-Mediated NDUFB6 Stabilization Regulated Mitochondrial Oxidative Phosphorylation
Source: Research (Wash D C). 2025 Dec 12;8:0985. doi: 10.34133/research.0985 (PMC13248703; doi:10.34133/research.0985)

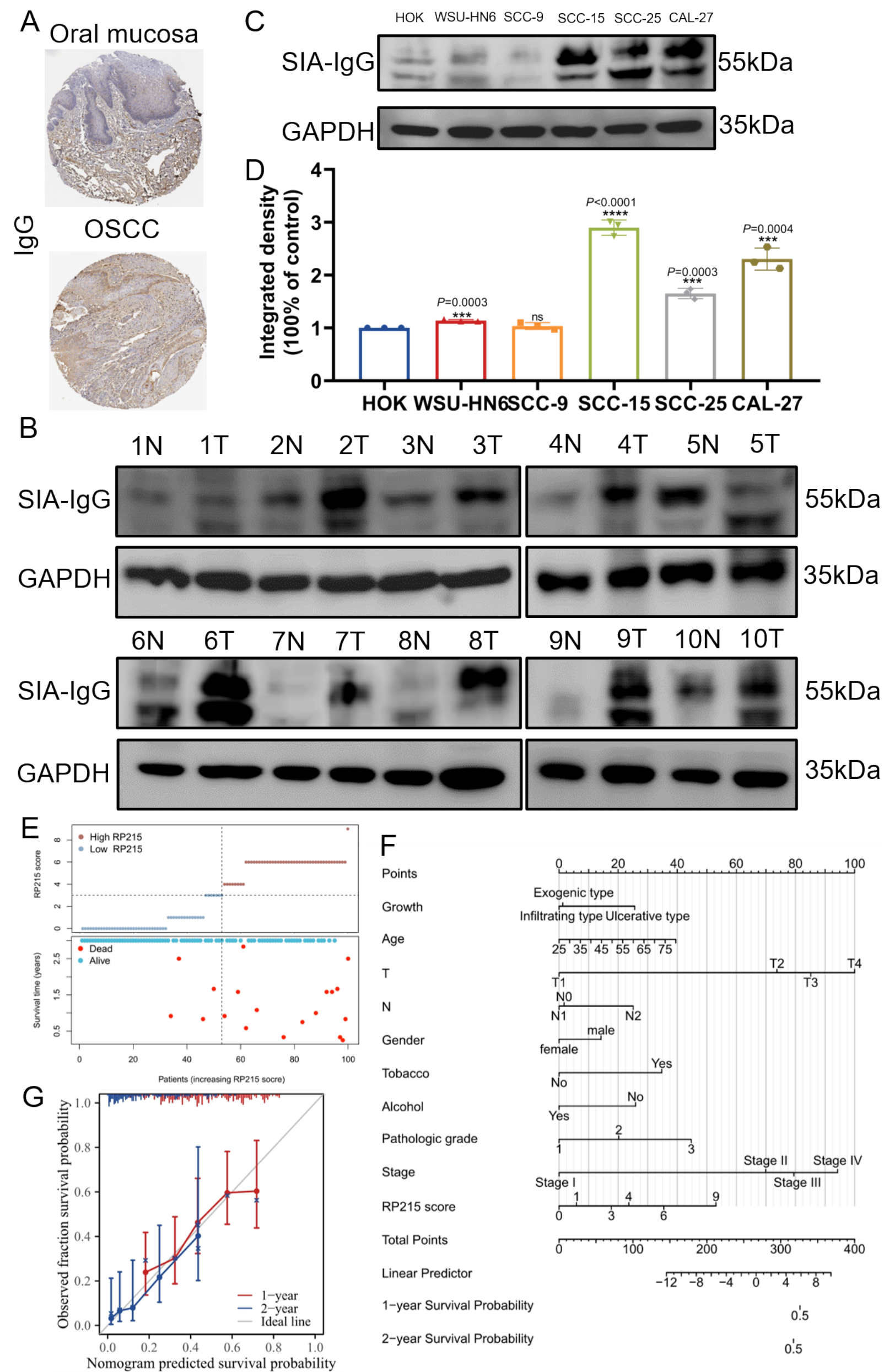

Supplement: Supplementary 1 — Figs. S1 to S11 [file research.0985.f1.zip › Figure S1.pdf]

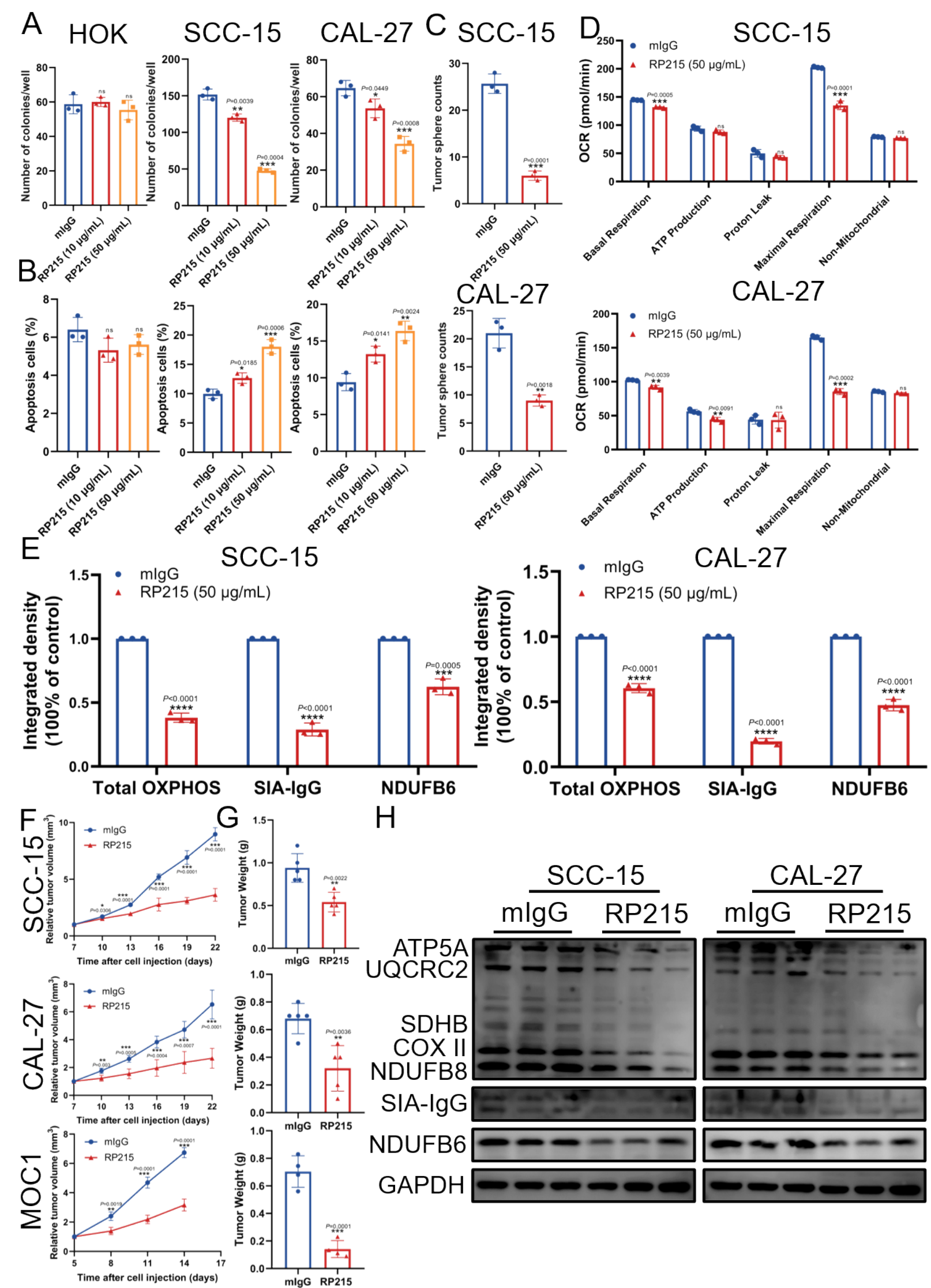

Supplement: Supplementary 1 — Figs. S1 to S11 [file research.0985.f1.zip › Figure S10.pdf]

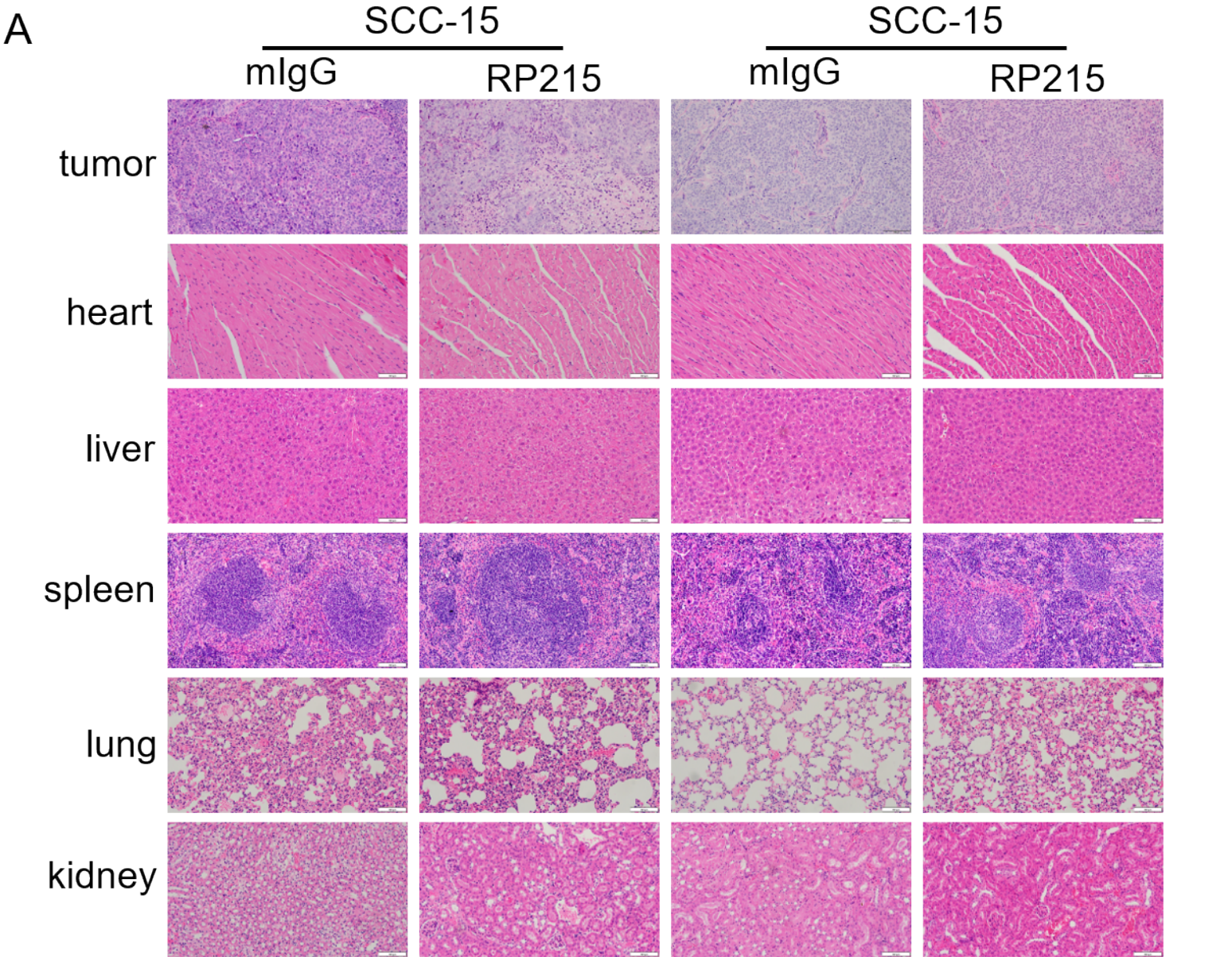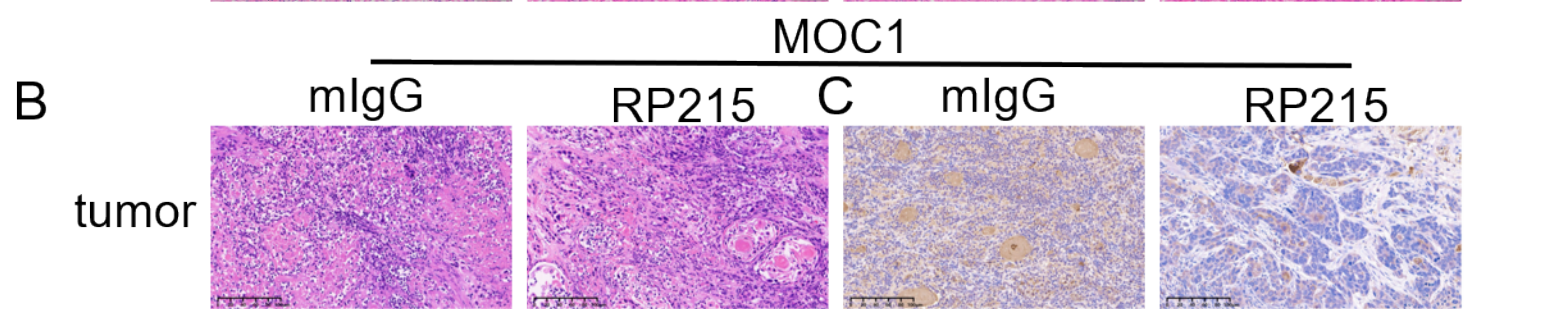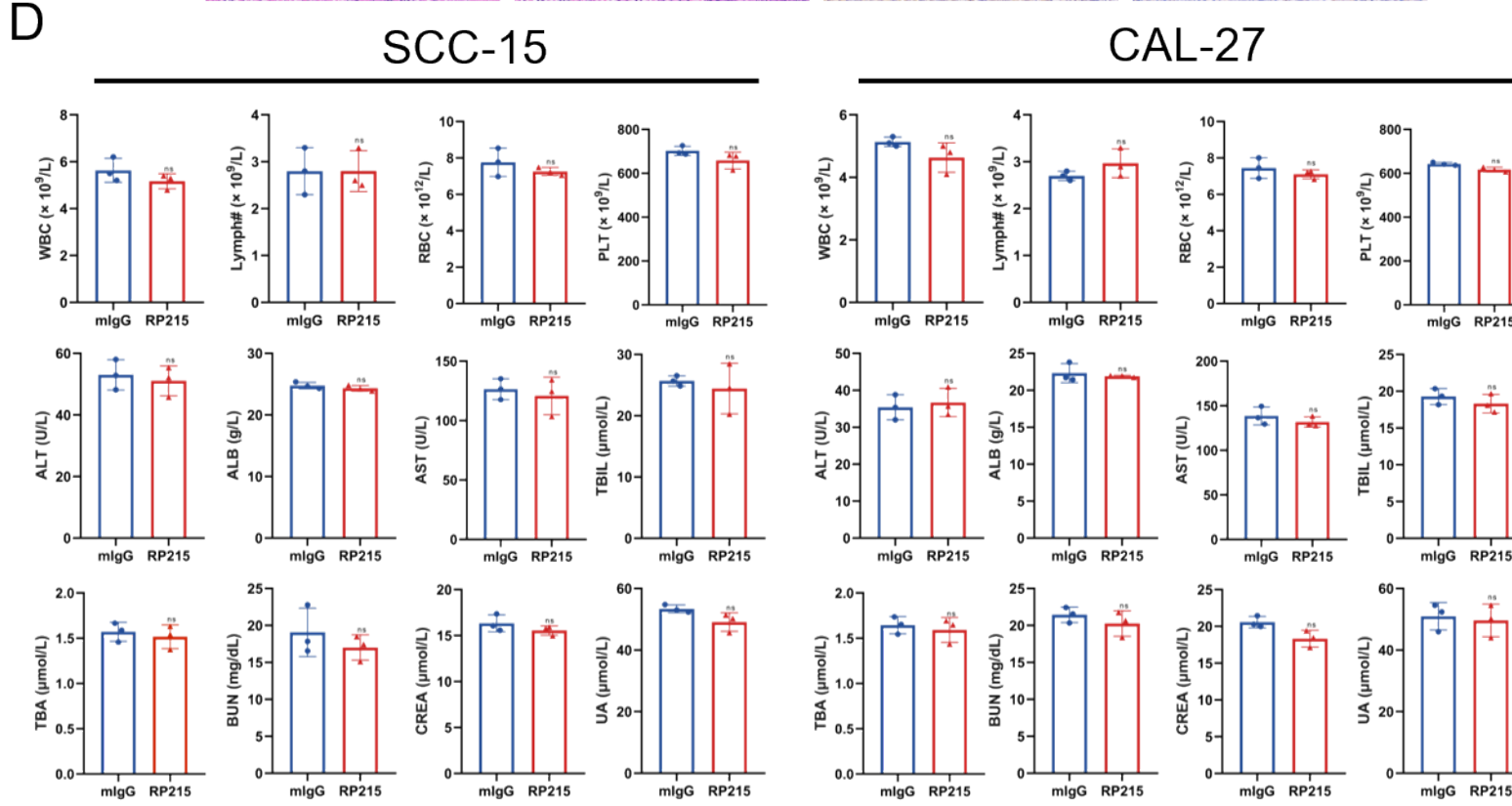

Supplement: Supplementary 1 — Figs. S1 to S11 [file research.0985.f1.zip › Figure S11.pdf]

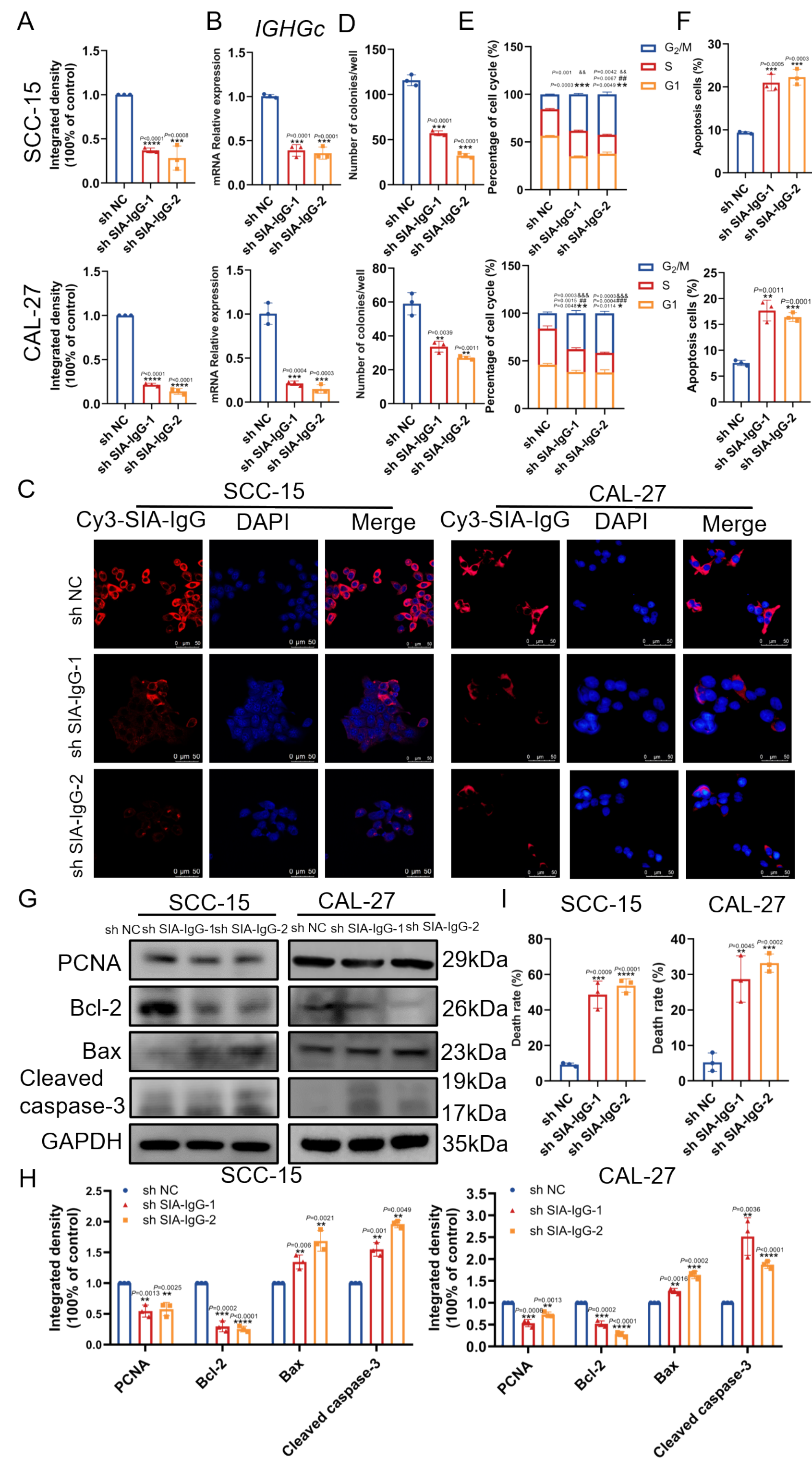

Supplement: Supplementary 1 — Figs. S1 to S11 [file research.0985.f1.zip › Figure S2.pdf]

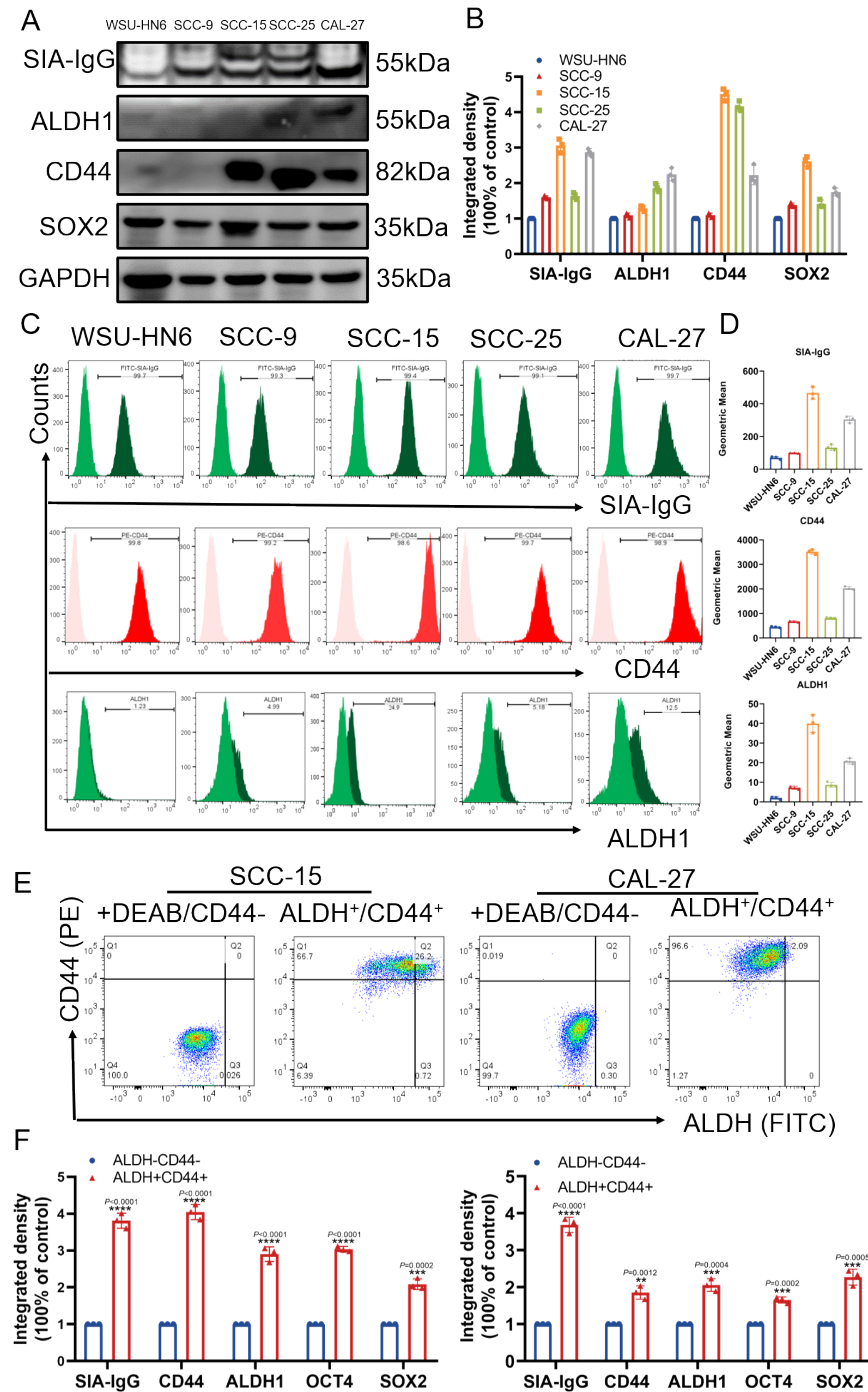

Supplement: Supplementary 1 — Figs. S1 to S11 [file research.0985.f1.zip › Figure S3.pdf]

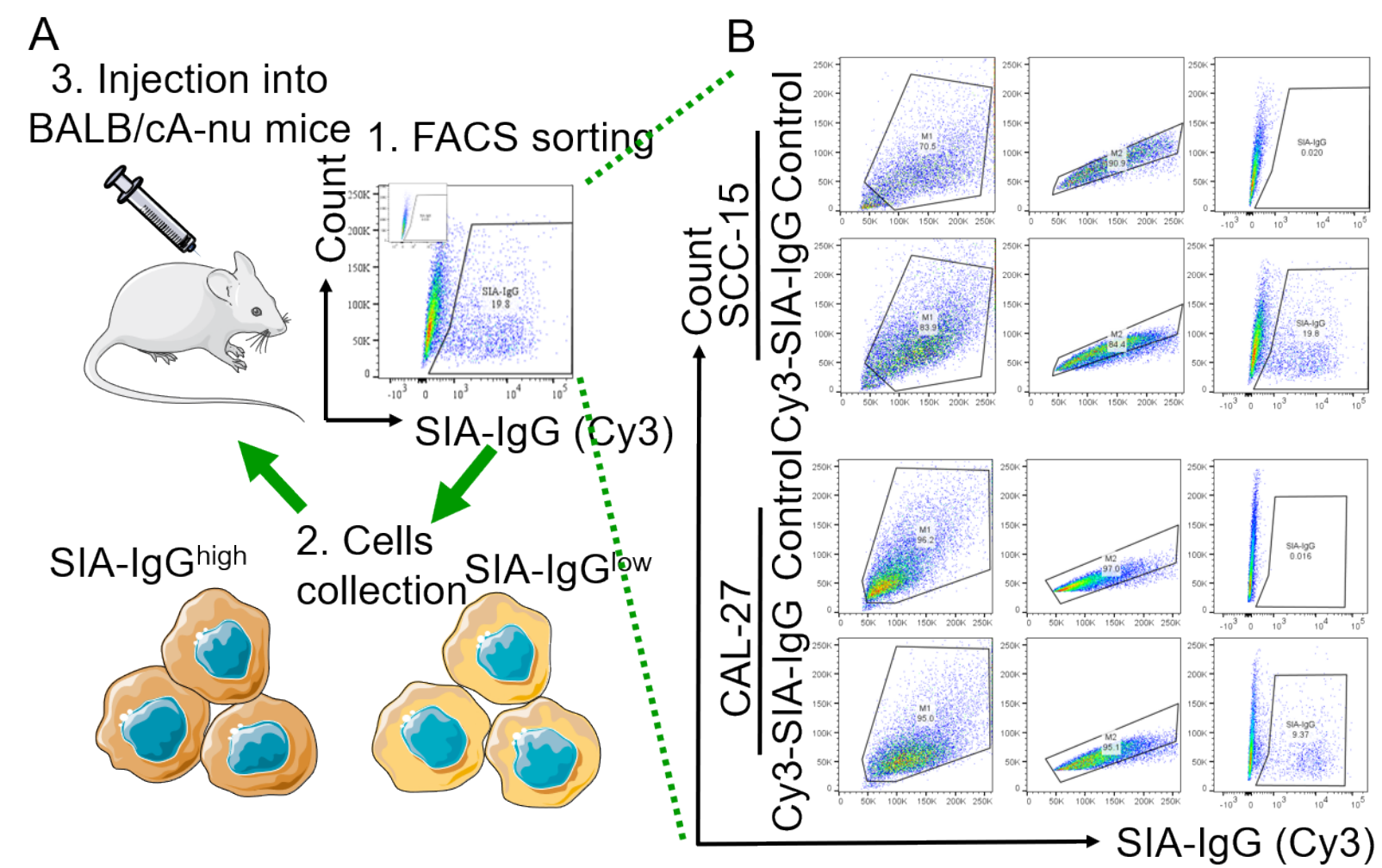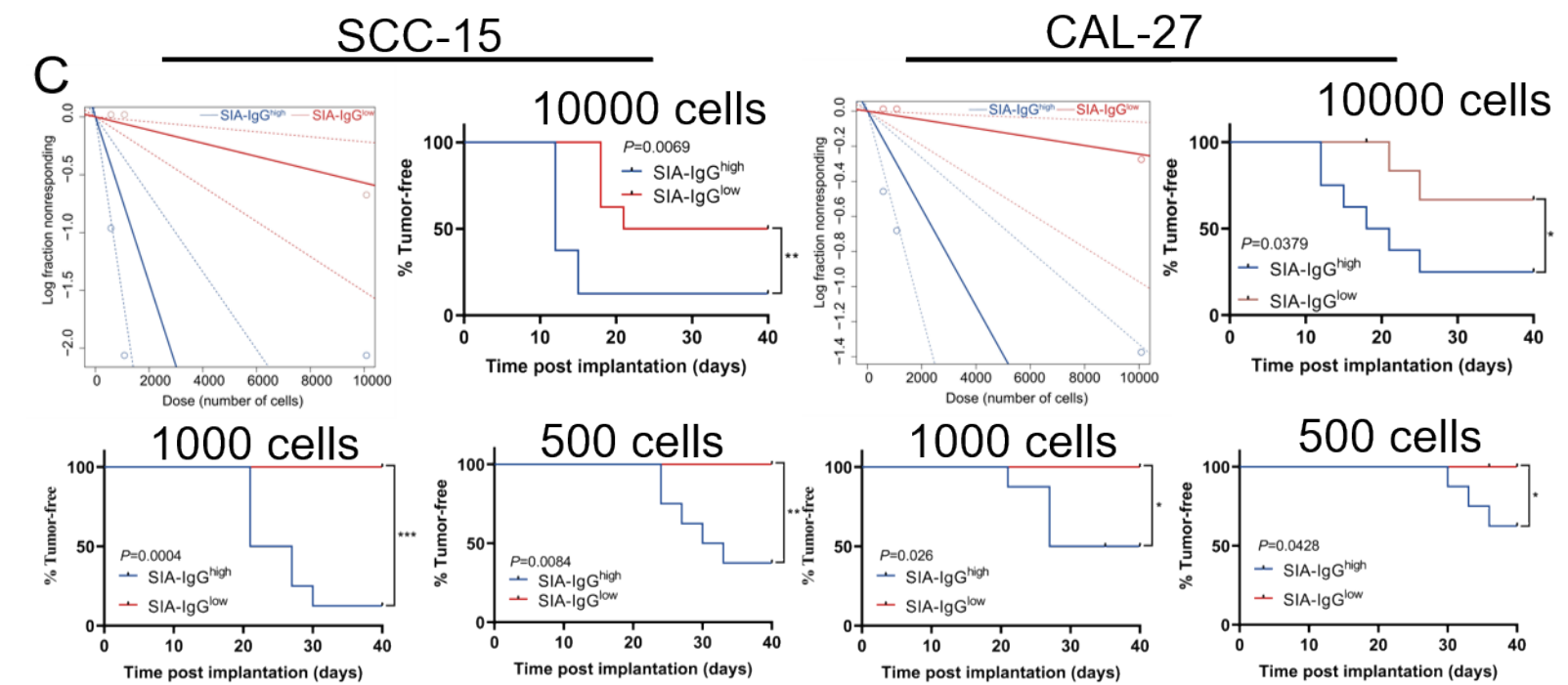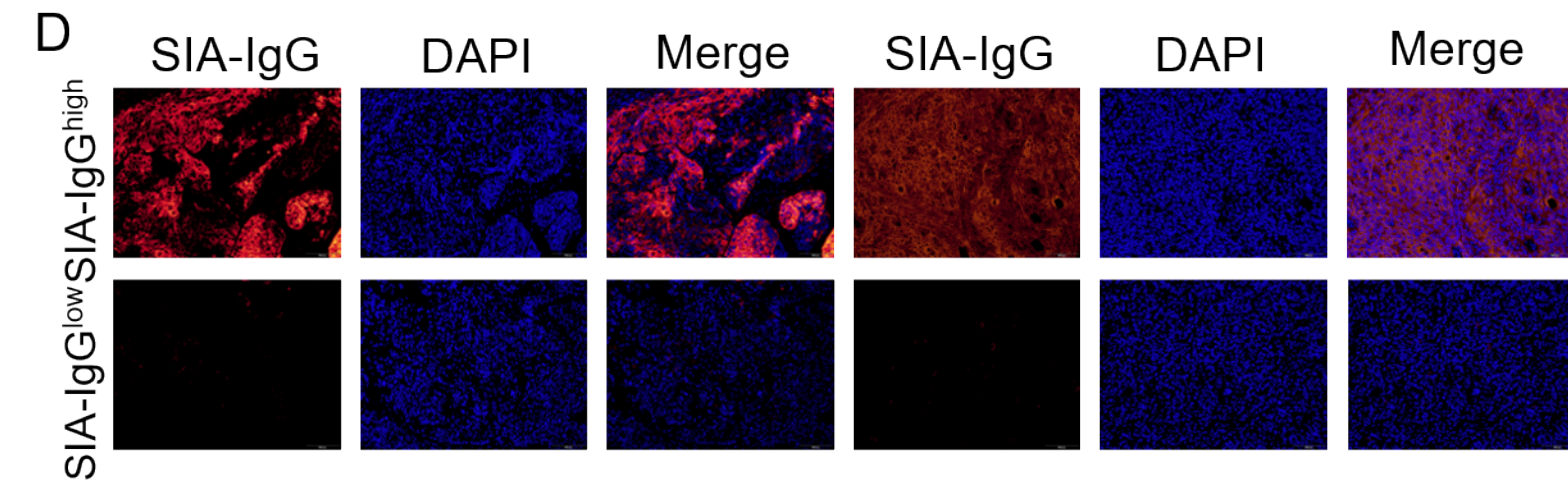

Supplement: Supplementary 1 — Figs. S1 to S11 [file research.0985.f1.zip › Figure S4.pdf]

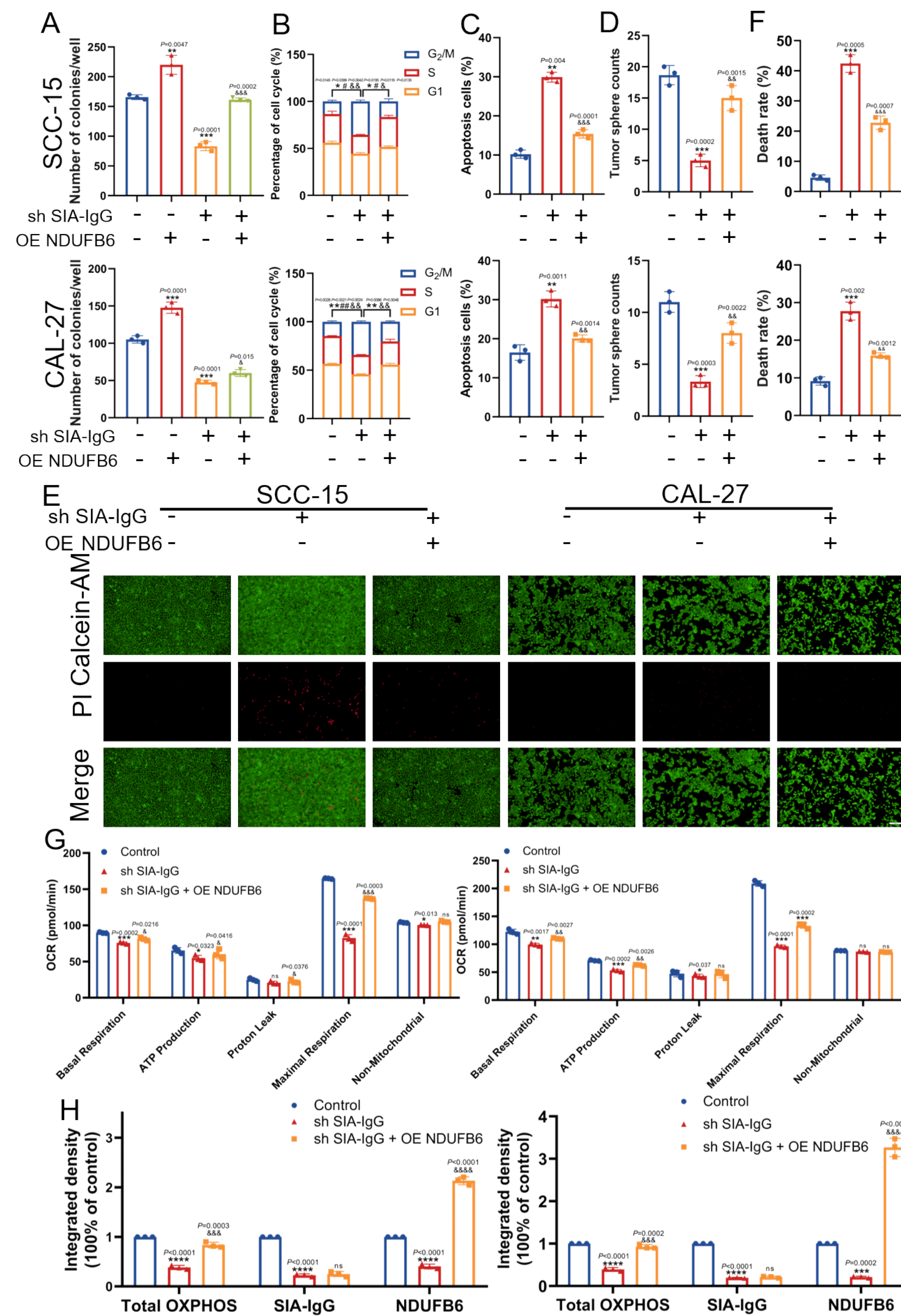

Supplement: Supplementary 1 — Figs. S1 to S11 [file research.0985.f1.zip › Figure S7.pdf]

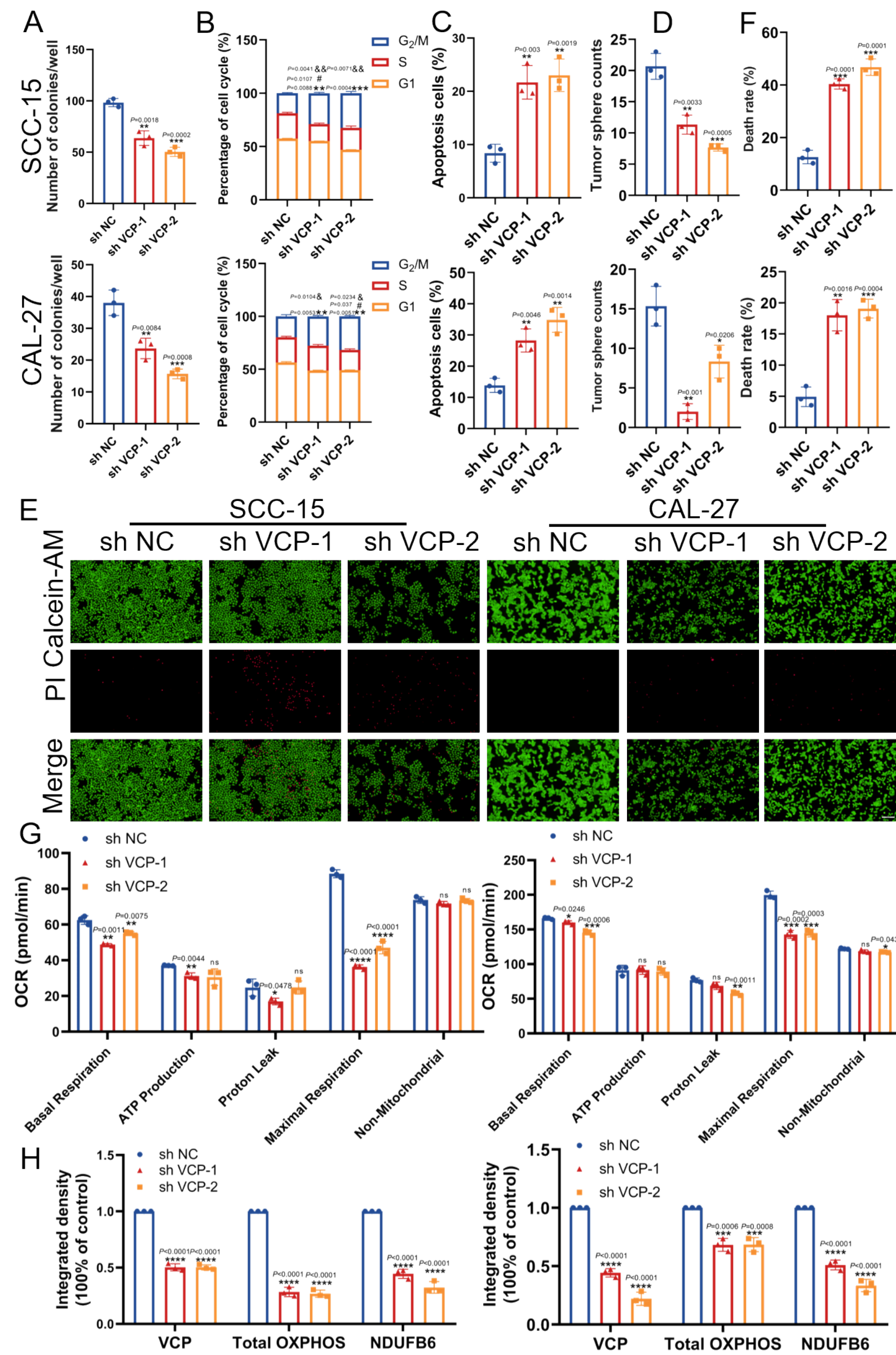

Supplement: Supplementary 1 — Figs. S1 to S11 [file research.0985.f1.zip › Figure S8.pdf]

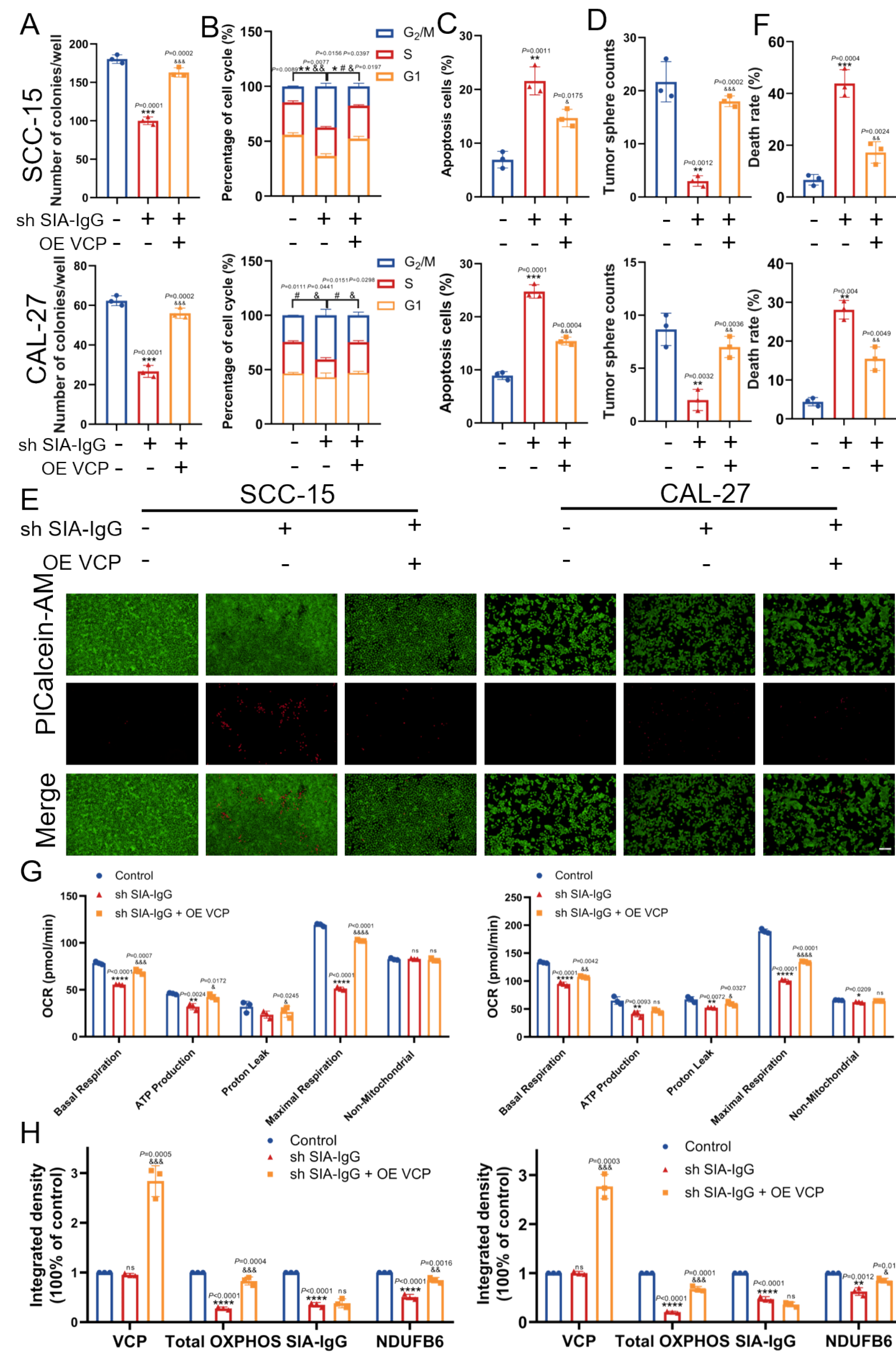

Supplement: Supplementary 1 — Figs. S1 to S11 [file research.0985.f1.zip › Figure S9.pdf]
